# Depictions of Depression in Generative AI Video Models: Mixed Methods Study of OpenAI’s Sora 2

**DOI:** 10.2196/95682

**Published:** 2026-09-14

**Authors:** Matthew Flathers, Griffin Smith, Julian Herpertz, Zhitong Zhou, John Torous

**Affiliations:** 1 Division of Digital Psychiatry, Beth Israel Deaconess Medical Center, 20 Overland St, Suite OV-202, Boston, MA, 02215, United States, 1 6176676700; 2 Digital + Media, Rhode Island School of Design, Providence, RI, United States; 3 Department of Psychiatry and Psychotherapy, Campus Benjamin Franklin, Charité – Universitätsmedizin Berlin, Berlin, Germany; 4 School of Public Health, Boston University, Boston, MA, United States

**Keywords:** artificial intelligence, AI, generative AI, depression, mental health representation, text to video, computer vision

## Abstract

**Background:**

Generative AI video models are increasingly capable of producing complex depictions of mental health experiences, yet little is known about how these systems represent conditions such as depression. Because AI-generated content may reach people during vulnerable periods, understanding what visual narratives these models produce for sensitive concepts carries clinical relevance.

**Objective:**

This study aimed to characterize how OpenAI’s Sora 2 generative AI video model depicts depression and examine whether depictions differ between the consumer app and developer API access points, which differ in their product layer mediation.

**Methods:**

We generated 100 videos using the single-word prompt “Depression” across 2 access points: the consumer app (n=50, 50%) and developer API (n=50, 50%). Two trained coders independently coded narrative structure, visual environments, objects, figure demographics, and figure states. Interrater reliability was assessed using the Cohen κ, with dimensions showing insufficient agreement excluded from analysis. Computational features (visual aesthetics, audio, semantic content, and temporal dynamics) were extracted and compared between modalities using 2-tailed Welch *t* tests with Benjamini-Hochberg false discovery rate correction.

**Results:**

App-generated videos exhibited a pronounced recovery bias: 78% (39/50) featured narrative arcs progressing from depressive states toward resolution compared with 14% (7/50) of API outputs. This divergence was reinforced across channels. App videos brightened over time (mean slope 2.90, SD 2.43 per second vs −0.18, SD 1.24 per second for the API; Cohen *d*=1.59; *q*<.001) and contained 3 times more motion (Cohen *d*=2.07; *q*<.001). Across both modalities, videos converged on a narrow visual vocabulary: predominantly seated figures (94/100, 94% of the videos); downward gaze (93/100, 93% of the videos); and recurring objects including hoodies (n=194), windows (n=148), and rain (n=83). Transcript language in depressive phases emphasized weight and containment (“heavy,” “drowning,” and “room”), whereas recovery phases reversed these patterns: brightness increased by 26.6% (Cohen *d*=0.68; *P*<.001), gaze shifted upward in 67% (30/45) of recovery videos, and terms such as “light” and “breath” emerged. Figures were predominantly young adults (323/367, 88% aged 20-30 years) and nearly always alone (360/367, 98%). Gender varied by access point: app outputs skewed male (34/50, 68%), and API outputs skewed female (33/56, 59%).

**Conclusions:**

Sora 2 does not invent new visual grammars for depression but compresses and recombines cultural iconographies, whereas platform-level constraints substantially shape which narratives reach users. Clinicians should be aware that AI-generated mental health video content reflects training data and platform design rather than clinical knowledge and that patients may encounter such content during vulnerable periods.

## Introduction

Media representations of psychiatric conditions shape how the public understands mental illness and how patients understand their own diagnoses [1-4]. Increasingly, these representations are circulating through short-form video (SFV) platforms. Mental health has emerged as one of the most prevalent topics on TikTok, Instagram Reels, and YouTube Shorts, with hashtags such as “#mentalhealth” accumulating over 44 billion views on TikTok alone [5]. SFV platforms have become embedded in daily life, with roughly 6 in 10 US teenagers visiting TikTok daily and one-third using at least one major platform almost constantly [6,7].

Against this backdrop, generative AI has introduced new capabilities for creating custom SFV content at scale. AI-generated videos can now be produced much faster and more cheaply than using traditional video editing pipelines. OpenAI’s Sora 2, released in September 2025, is an early leader in this space [8]. Within 72 hours of launch, the Sora mobile app became the top-downloaded app on the iOS App Store, surpassing ChatGPT with over 164,000 downloads [9]. The app functions as a social network for AI-generated videos, featuring a vertical feed of 10-second clips with likes, comments, and remix tools [8]. In December 2025, OpenAI announced a partnership with Disney that will bring over 200 characters from Disney, Marvel Studios, Pixar, and the *Star Wars* franchise to the platform [10]. OpenAI’s policies prohibit use by children under 13 years [11], yet licensing characters from franchises with young audiences creates obvious pressure on these boundaries. As these capabilities expand beyond entertainment, AI videos will increasingly reach people who are seeking to understand their own mental health.

Sora 2 is not the only platform. Multiple laboratories have released generative AI video models that can produce highly realistic outputs that viewers cannot always reliably distinguish from authentic footage [12]. Google’s Veo 3 pairs high-fidelity video generation with native audio generation [13], Runway’s Gen-4.5 provides sophisticated video editing capabilities [14], Meta AI has launched Vibes to integrate AI video generation into its social ecosystem [15], and ByteDance’s Seedance demonstrates high-fidelity multi-shot video generation with consistent subject representation across scenes [16]. Most generative AI video tools build on diffusion image model architectures, much like AI image models. Unlike transformer-based language models that predict and generate words sequentially, diffusion models learn to transform noise into coherent imagery. They do this through a two-phase process: systematically adding noise to images until they become visual static (forward diffusion) and then learning to reverse this process to reconstruct coherent images [17,18]. Through training on billions of examples, these models learn the contours, patterns, and statistical regularities of their training data, storing this information across vast parameter spaces. Video diffusion models extend this into higher-dimensional space, maintaining consistency across hundreds of frames while generating plausible motion and transitions [19,20].

The outputs of these systems occupy a unique categorical space [21]. The underlying models are trained on large, proprietary datasets that presumably contain heterogeneous sources: clinical documentation, licensed stock footage, pharmaceutical advertisements, video blogs, and other user-generated content. The resulting synthesis reveals aggregate cultural understanding rather than any single authoritative source. When a user prompts a model with “Depression,” the resulting artifact is not summoned from nothing, nor is it copied from some original human source. The user’s prompt is mediated by a technical apparatus that constrains what can be produced to what its programming makes possible [22]. The output is “found” by the AI model in the diffusion process, selected from the space of possible outputs that training made probable. Whether the patterns learned during training extend beyond what things look like to capture implicit associations, co-occurrences, and cultural conventions embedded in the training data (as has been observed in image [23], text [24], and embedding [25] models) remains an open question.

**Figure 1.**
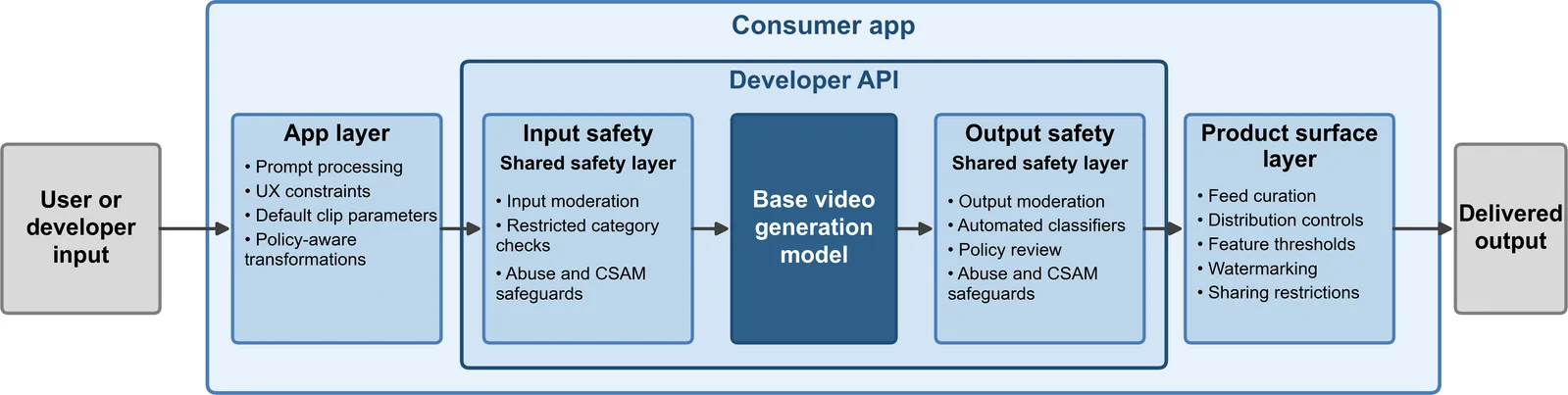
Generalized system architecture for generative AI video platforms, illustrating the distinction between the developer API and consumer app access pathways. Both pathways share core safety infrastructure (center), including input and output moderation. Consumer apps additionally route prompts through an app layer (prompt processing, user experience [UX] constraints, default clip parameters, and policy-aware transformations) before generation and through a product surface layer (feed curation, distribution controls, feature thresholds, watermarking, and distribution controls) after generation. The developer API bypasses these layers, accessing only the shared safety infrastructure and base model. CSAM: child sexual abuse material.

This study examined the Sora 2 generative AI video system, but our methodological approach addresses a broader issue in clinical AI research. Much existing work treats “the model” as the object of study without distinguishing between (1) the underlying models and (2) the products and consumer-facing apps and tools built upon them. When a user submits a prompt into the Sora app, they interact with a system that includes the model plus product layers. When a researcher accesses the same model through the API, fewer of these layers intervene (see Figure 1 for a generalized illustration of this distinction). Patterns observed in an app may therefore reflect product-level decisions (eg, more safety features and filters) rather than model-level associations, or vice versa. Studying only the product tells us what users encounter but not what the model has learned; studying only the model tells us what the training has encoded but not what reaches users [26]. Comparing both access points reveals something about the safety and content moderation layers that companies have implemented for sensitive topics such as depression. The distinction between model and product is also important for research on safety evaluation within the wider AI product ecosystem. Future developers will build applications on top of AI models accessed via APIs, not the consumer product layer. Sora 2 provides an opportunity to examine these dynamics because OpenAI has made both access points available: the consumer app and the developer API.

When someone experiencing a depressive episode searches for content about depression and is served AI-generated videos on platforms such as Sora 2, those outputs become part of their informational environment during a vulnerable period. The content might reinforce their experience, normalize or stigmatize it, or offer implicit models of recovery or self-harm [4,27,28]. Whether AI-generated depictions of depression draw on clinical understanding, popular media tropes, pharmaceutical advertising, or some mixture remains an empirical question. Addressing this question has important implications for how clinicians think about the information environment surrounding their patients. As a first exploratory step, we asked what patterns Sora 2 produces for the unqualified prompt “Depression” and whether these differ between the consumer app and developer API.

## Methods

### Data Generation

We generated 50 videos using each of 2 access points to OpenAI’s Sora 2 system (the consumer-facing mobile app and the developer API), yielding a total corpus of 100 videos. All videos were generated in portrait orientation (9:16 aspect ratio) with each platform’s standard output parameters: 10-second clips at 720p resolution from the app and 12-second clips at 720p resolution using the Sora 2 end point (sora-2) via the OpenAI Responses API. To control for potential model updates, all generations occurred within a single 1-week window (November 21‐28, 2025). All app videos were generated from a new Sora account with no prior video generation history.

Each video was generated using the isolated prompt “Depression” with no additional qualifiers, contextual framing, or prompt engineering. Our minimal prompt design was a deliberate methodological choice. The prompt “Depression” is inherently ambiguous. It might refer to a clinical mood disorder, a colloquial feeling, a topographic feature, or an economic phenomenon. Elaborate prompts resolve this ambiguity for the model; minimal prompts force the model to resolve it from its encoded associations. A prompt such as “a depressed person sitting alone in a dark room crying” tests whether a model can render a specified scene but reveals little about what the model associates with depression as a concept because the user has already supplied the interpretive content. Single-term prompts shift this interpretive labor to the model. When given only “Depression” with no contextual elaboration, the model must supply setting, figures, mood, color, motion, and narrative entirely from its encoded associations. Every visual choice in the output, therefore, reflects training data or product-level steering rather than user instruction, making minimal prompting a more sensitive probe of the model’s default representations.

### Qualitative Analysis

Two authors (MF and ZZ) independently conducted a qualitative content analysis of all videos using a directed approach in which initial coding categories were derived from prior literature and the study’s research questions [29]. Each coder tagged videos across six dimensions: (1) narrative arc (presence and direction of emotional shifts), (2) environments (physical settings with time stamps), (3) objects (visual symbols and props, with time stamps), (4) figure demographics (apparent gender, race and ethnicity, and age, inferred from visual appearance and treated as perceived and not ground truth), (5) figure states (posture, facial expression, and being alone in the frame, with time stamps), and (6) overall notes (AI artifacts and uncertainties). Raw codes were harmonized to standardized categories prior to analysis. Extended coding procedures and dimension rationale can be found in Multimedia Appendix 1, the full codebook can be found in Multimedia Appendix 2, and harmonization dictionaries can be found in Multimedia Appendix 3.

### Reliability

Both authors independently coded the full dataset. One author’s coding (MF) was designated as the primary dataset for analysis; the second author’s coding (ZZ) served as interrater reliability verification. The Cohen κ was calculated for the presence or absence of categorical elements and for dimensions involving temporal segments, and we also calculated intersection over union at 1-second resolution to assess agreement on timing. Given the scale of the dataset, item-by-item consensus resolution was not feasible; reliability metrics verified that the coding scheme could be applied consistently across coders.

### Quantitative Analysis

To complement qualitative coding, we extracted computational features across 4 domains: visual aesthetics, audio properties, semantic content, and temporal dynamics. Visual features included brightness, saturation, colorfulness, color temperature, and gray-level co-occurrence matrix texture measures [30] sampled at 1-second intervals. Audio analysis extracted volume, pitch, and spectral centroid using the librosa library [31]. Semantic analysis of transcribed speech (OpenAI Whisper [32]) included Valence Aware Dictionary and Sentiment Reasoner sentiment [33], custom light and dark lexicons based on conceptual metaphor theory [34], and linguistic markers including first-person pronoun ratio and negation frequency. Temporal dynamics were captured through dense optical flow (Farnebäck algorithm [35]), scene detection, and per-second feature trajectories. Speech-dependent features were computed only for videos with detectable speech, whereas all videos were included in the visual, texture, motion, and audio analyses. Features were selected based on documented connections to depression representation in prior research; technical specifications and literature rationale can be found in Multimedia Appendix 4.

### Ethical Considerations

This study analyzed AI-generated video content produced by a commercially available system (OpenAI Sora 2) and did not involve human participants, human biological materials, or identifiable personal data. No human participants were recruited, surveyed, or observed, and no personally identifiable information was collected or analyzed. Accordingly, institutional review board review was not required under federal regulations (Title 45 of the Code of Federal Regulations part 46) or institutional policy, and informed consent and participant compensation were not applicable. All analyzed content was generated by the research team using publicly available AI tools.

### Statistical Analysis

For each quantitative feature, we computed the mean and SD for each access modality. Differences between app and API outputs were assessed using two-tailed Welch *t* tests [36], with Benjamini-Hochberg false discovery rate (FDR) correction [37] to control for multiple comparisons across all 28 features tested (full specifications can be found in Multimedia Appendix 4). Effect sizes were quantified using the Cohen *d* [38]. To characterize temporal dynamics, we computed the linear slope of each feature’s time series and compared slope distributions between modalities using the same inferential approach.

## Results

### Quantitative Findings

#### Visual Aesthetics

**Figure 2.**
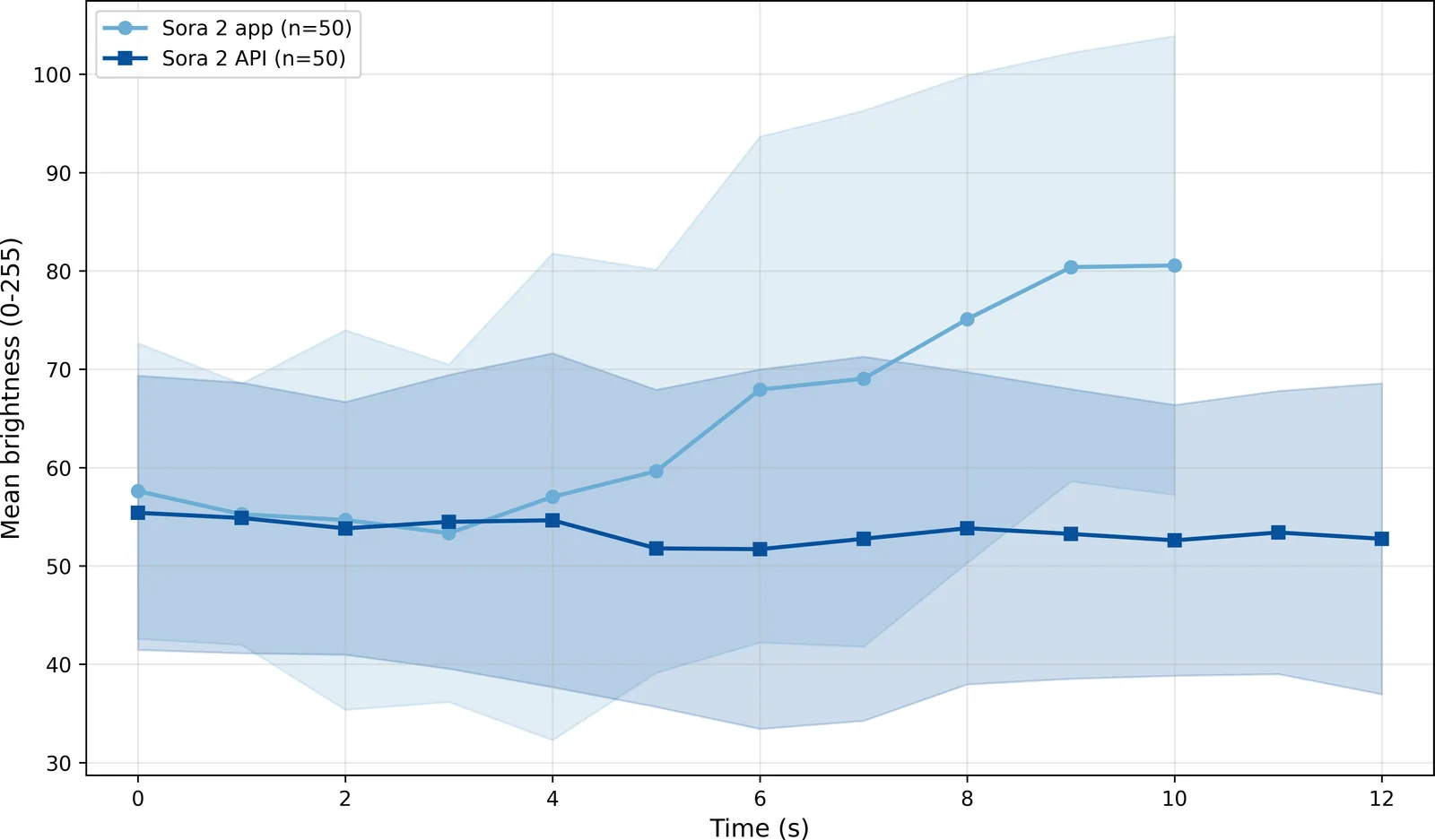
Brightness trajectory over time. Mean brightness (scale from 0‐255) at each second for app-generated (light blue; n=50) and API-generated (dark blue; n=50) videos. Shaded regions indicate the SD. App videos exhibit a pronounced brightening trajectory (mean slope 2.90, SD 2.43 per second), whereas API videos remain relatively flat (mean slope −0.18, SD 1.24 per second). The difference in brightness slope was statistically significant (Cohen *d*=1.59; *q*<.001; Welch *t* test with Benjamini-Hochberg false discovery rate correction).

App-generated videos were significantly brighter than API videos (mean 64.59, SD 13.35 vs 53.48, SD 11.86; Cohen *d*=0.87; *q*<.001) (Table 1). This difference emerged over time: app videos exhibited a pronounced brightening trajectory (mean slope 2.90, SD 2.43 per second), whereas API videos remained essentially flat (mean slope −0.18, SD 1.24 per second; Cohen *d*=1.59; *q*<.001). Figure 2 illustrates this divergence, showing app videos beginning at comparable brightness levels to those of API videos but progressively lightening across their duration.

**Table 1.** Aggregate descriptive statistics.

Category and feature	App (n=50), mean (SD)	API (n=50), mean (SD)	Cohen *d*	FDR^a^-corrected *q* value^b^
Visual				
Brightness (0‑255)	64.59 (13.35)	53.48 (11.86)	0.87	<.001^c^
Saturation (0-255)	99.24 (26.61)	92.48 (23.08)	0.27	.34
Colorfulness (dimensionless)	25.67 (7.48)	22.41 (8.01)	0.42	.09
Color temperature (dimensionless)	−13.38 (10.56)	−14.45 (9.66)	0.10	.72
Edge density (proportion)	0.03 (0.01)	0.03 (0.01)	−0.31	.24
Texture				
GLCM^d^ contrast (gray levels²)	12.15 (5.77)	7.78 (5.11)	0.79	<.001^c^
GLCM dissimilarity (gray levels)	1.20 (0.33)	1.12 (0.31)	0.22	.45
GLCM homogeneity (0-1)	0.72 (0.05)	0.70 (0.05)	0.51	.03^c^
GLCM energy (0-1)	0.17 (0.04)	0.17 (0.04)	−0.03	.91
GLCM correlation (-1 to 1)	0.96 (0.02)	0.97 (0.02)	−0.53	.03^c^
GLCM entropy (bits)	6.98 (0.53)	6.94 (0.51)	0.09	.73
Motion				
Optical flow (pixels per frame)	0.35 (0.15)	0.11 (0.07)	2.07	<.001^c^
Scene cut count (count)	3.10 (1.88)	0.76 (1.42)	1.39	<.001^c^
Audio				
Volume (RMS^e^, 0-1)	0.10 (0.01)	0.09 (0.01)	0.83	<.001^c^
Pitch (Hz)	115.51 (30.04)	148.87 (40.78)	−0.92	<.001^c^
Spectral centroid (Hz)	1744.08 (395.02)	1792.82 (284.42)	−0.14	.63
Speech				
Word count (count)	31.40 (5.70)	36.50 (8.01)	−0.73	.001^c^
Speech rate (words per s of speech)	4.18 (0.64)	4.07 (0.54)	0.19	.55
Sentiment				
Compound (-1 to 1)	0.19 (0.33)	0.24 (0.36)	−0.15	.63
Positive (0-1)	0.09 (0.05)	0.10 (0.07)	−0.17	.55
Negative (0-1)	0.04 (0.05)	0.04 (0.04)	−0.07	.78
Neutral (0-1)	0.87 (0.06)	0.86 (0.08)	0.18	.55
Semantic				
Light word count (count)	0.02 (0.14)	0.16 (0.37)	−0.50	.04^c^
Dark word count (count)	0.58 (0.67)	0.64 (0.52)	−0.10	.72
Light-to-dark word ratio (0-1)	0.27 (0.27)	0.27 (0.29)	0.00	>.99
First-person pronoun ratio (0-1)	0.04 (0.04)	0.08 (0.05)	−0.78	.001^c^
Negation ratio (0-1)	0.02 (0.03)	0.03 (0.03)	−0.12	.70
Lexical diversity (0-1)	0.90 (0.05)	0.89 (0.05)	0.22	.45

a FDR: false discovery rate.

b two-tailed Welch *t *test with Benjamini-Hochberg correction.

c *q*<.05.

d GLCM: gray-level co-occurrence matrix.

e RMS: root mean square.

Chromatic properties showed complementary patterns. Although mean saturation did not differ significantly between modalities (*q*=.34), saturation trajectories diverged substantially: app videos desaturated over time (mean slope −2.99, SD 3.17 per second), whereas API videos maintained stable saturation (mean slope 0.24, SD 1.30 per second; Cohen *d*=−1.33; *q*<.001). Similarly, app videos warmed in color temperature over time (mean slope 2.24, SD 2.26 per second) compared to minimal change in API videos (mean slope 0.18, SD 0.85 per second; Cohen *d*=1.21; *q*<.001). Together, these trajectories describe a characteristic app visual arc: beginning in cool, saturated tones and transitioning toward warm, desaturated, brighter imagery.

Texture analysis revealed limited additional differences. App videos exhibited higher gray-level co-occurrence matrix contrast (mean 12.15, SD 5.77 vs 7.78, SD 5.11; Cohen *d*=0.79; *q*=.001) and homogeneity (Cohen *d*=0.51; *q*=.03), indicating sharper local intensity variation and more uniform textural regions. Other texture features remained largely consistent across modalities.

#### Temporal Dynamics

**Figure 3.**
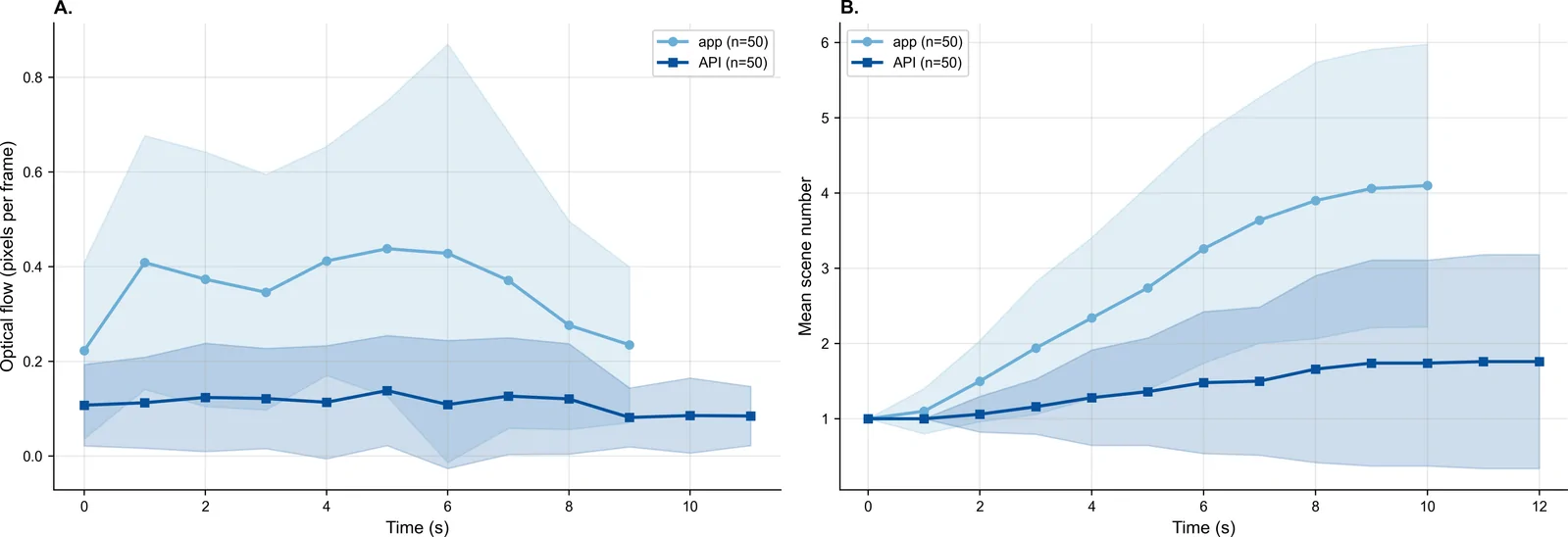
Temporal dynamics of motion and editing in app vs API videos (n=50 per access point). (A) Motion intensity: mean optical flow magnitude (pixels per frame; Farnebäck dense optical flow) at each second. (B) Scene accumulation: mean cumulative scene number at each second, where scene 1 denotes the opening scene and each cut increments the count by 1. Shaded bands indicate the SD.

The most pronounced differences emerged in motion characteristics. App videos contained approximately 3 times more optical flow than API videos (mean 0.35, SD 0.15 vs 0.11, SD 0.07 pixels per frame; Cohen *d*=2.07; *q*<.001), indicating substantially greater movement within scenes (Figure 3A). This difference persisted across the full video duration, with app videos maintaining elevated motion throughout rather than concentrating movement in particular segments.

Scene structure differed dramatically between modalities. App videos contained a mean of 3.10 (SD 1.88) scene cuts, whereas API videos contained only 0.76 (SD 1.42) cuts (Cohen *d*=1.39; *q*<.001). Figure 3B visualizes this difference as scene accumulation over time: by the end of app videos, viewers had encountered approximately 4 distinct scenes on average compared to fewer than 2 for API videos.

#### Audio and Speech

Audio properties diverged across modalities. App videos were slightly louder (mean 0.10, SD 0.01 vs 0.09, SD 0.01; Cohen *d*=0.83; *q*<.001) but featured lower-pitched audio (mean 115.51, SD 30.04 Hz vs 148.87, SD 40.78 Hz; Cohen *d*=−0.92; *q*<.001). Lower pitch in app videos may reflect the predominance of male narrators, different ambient sound profiles, or compositional choices in generated music.

Speech content also differed. API videos used first-person pronouns at nearly twice the rate of app videos (mean 0.08, SD 0.05 vs 0.04, SD 0.04; Cohen *d*=−0.78; *q*=.001). API videos also contained more light-associated words (mean 0.16, SD 0.37 vs 0.02, SD 0.14; Cohen *d*=−0.50; *q*=.04). Sentiment valence did not differ significantly between modalities (*q*=.63), with both producing mildly positive compound sentiment scores (app: mean 0.19, SD 0.33; API: mean 0.24, SD 0.36).

#### Semantic Content

**Figure 4.**
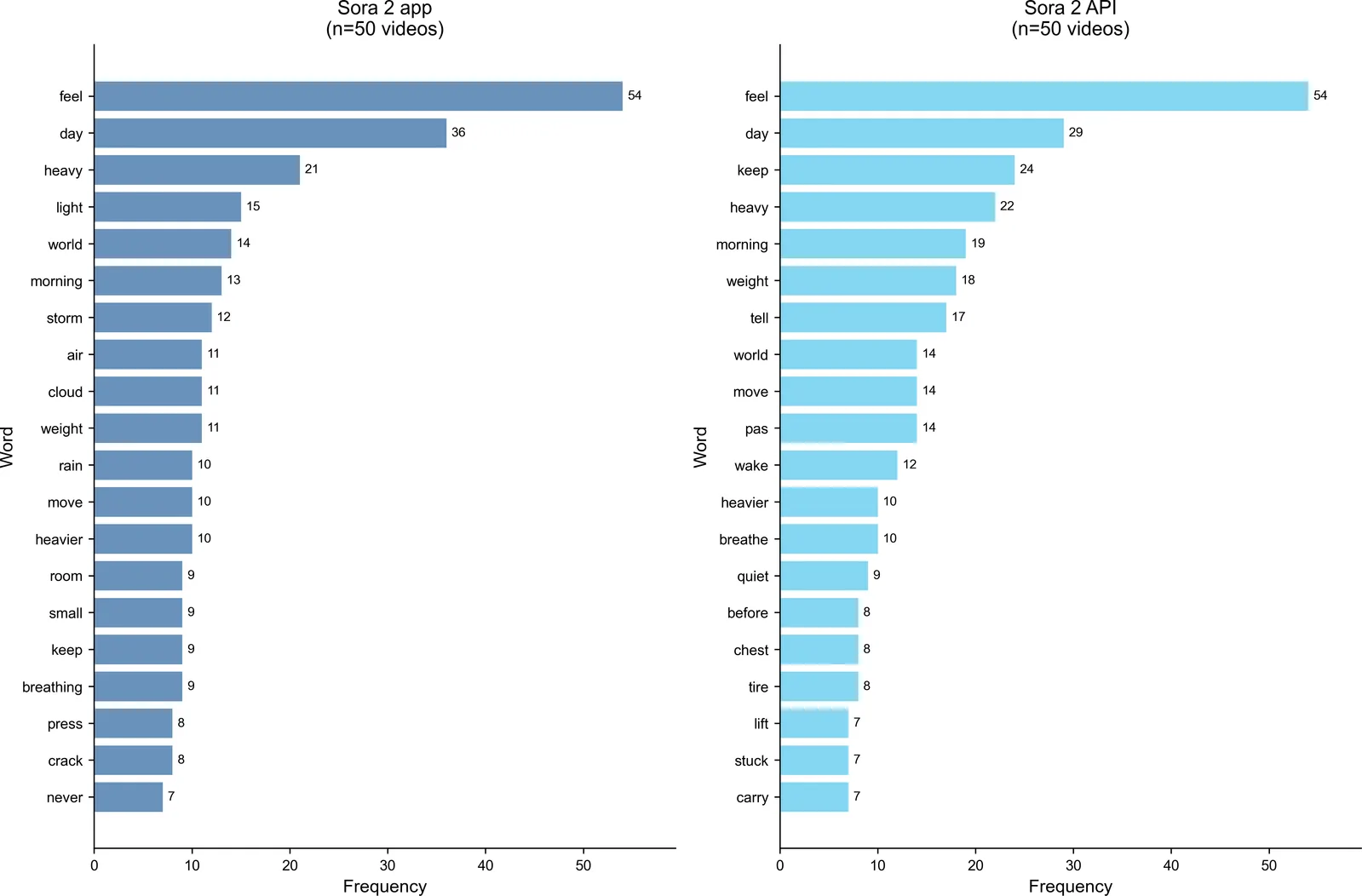
Word frequency comparison. Twenty most frequent lemmatized content words appearing in transcribed speech across app- and API-generated videos. Common terms cluster around themes of emotional experience (“feel,” “heavy,” and “weight”), temporal struggle (“day” and “morning”), natural metaphors (“storm,” “rain,” and “cloud”), and hope or release (“light” and “breathe”). This vocabulary reflects culturally prevalent metaphors for depression emphasizing weight, weather, darkness, and the possibility of relief.

Word frequency analysis revealed a coherent thematic vocabulary across the corpus (Figure 4). The most frequent terms clustered around emotional experience (“feel”: n=108; “heavy”: n=43), temporal struggle (“day”: n=65; “morning”: n=32), natural metaphors (“storm”: n=15; “rain”: n=17; “cloud”: n=12), and hope or release (“light”: n=19; “breathe”: n=14).

### Qualitative Findings

#### Interrater Reliability

Two coders independently coded all videos. Reliability was strong for most dimensions (narrative arc: κ=0.71; environments: κ=0.91 and intersection over union=0.84; posture: κ=0.89; being alone in the frame: κ=0.94; gender: κ=1.00). Object coding showed moderate overall agreement (κ=0.61) with variation by object type. Dimensions with insufficient reliability were excluded from analysis (facial expression: κ=0.49; race and ethnicity: κ=0.53; apparent socioeconomic status: κ=0.20). For objects, lower κ values for ubiquitous items such as beds (κ=0.44) and windows (κ=0.47) reflected asymmetric exhaustiveness between coders rather than substantive disagreement on object presence; the primary coder tagged these common items more exhaustively, whereas the secondary coder focused on more distinctive elements. Full reliability results can be found in Multimedia Appendix 5.

#### Narrative and Visual Patterns

**Figure 5.**
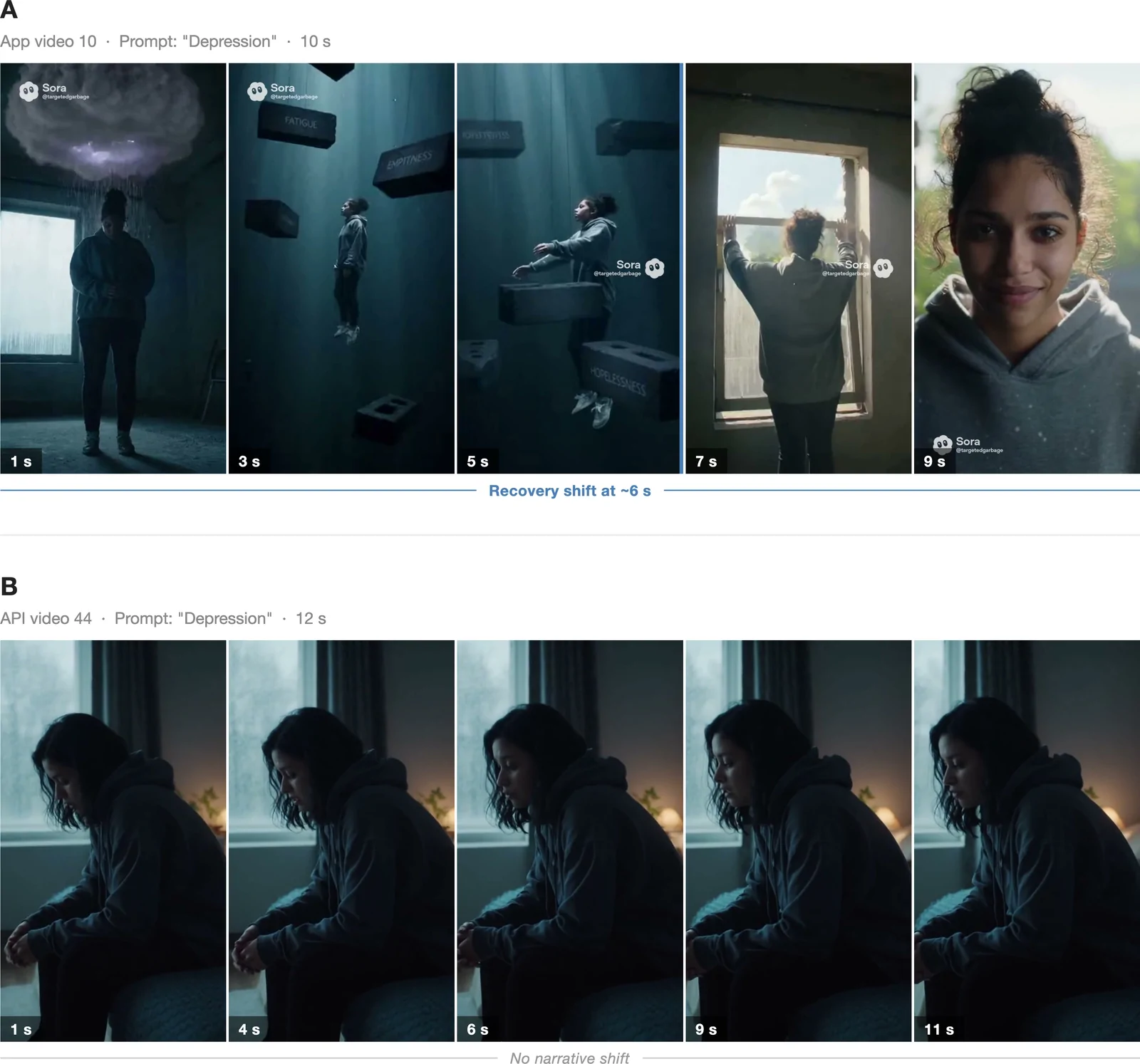
Representative video stills from app and API outputs generated using the prompt “Depression.” (A) Consumer app video showing a typical recovery arc. The video progresses from a dark interior with a personal storm cloud (1 second) to floating debris labeled with terms such as “fatigue” and “hopelessness” (3‐5 seconds) to the figure turning toward a bright window (7 seconds) and ending outdoors in sunlight, smiling (9 seconds). The coded recovery shift occurs at approximately 6 seconds. (B) Developer API video showing typical stasis. The figure remains seated on a bed in a dim bedroom throughout the full 12-second duration, wearing a gray hoodie with downward gaze; clasped hands; and minimal change in posture, lighting, or environment. Both videos were generated using identical single-word prompts and no additional parameters.

Despite the prompt’s lexical ambiguity, all videos depicted the affective sense of depression (eg, figures in emotional distress in personal settings) rather than its economic or topographic senses. The most striking qualitative finding concerned narrative structure. After collapsing to binary classification (recovery vs no recovery, combining deterioration and no shift), a dramatic divergence emerged between modalities. App videos frequently depicted recovery narratives, with 78% (39/50) of the videos containing a discernible shift toward hope or relief. API videos showed the opposite pattern: only 14% (7/50) of the videos depicted recovery arcs (Figure 5). This aligns with quantitative trajectory analysis showing app videos brightening over time, whereas API videos remained flat.

Across both modalities, videos converged on a narrow visual vocabulary. The bedroom was the dominant setting, with app videos featuring somewhat more diverse environments, including elevated outdoor spaces and urban settings. The most common objects were hoodies (n=194), windows (n=148), beds (n=114), rain (n=83), and lamps (n=59).

Generated figures were predominantly young adults aged 20 to 30 years (323/367, 88% of coded figure states) and nearly always alone in the frame (360/367, 98% of coded figure states). By far the dominant posture was sitting, present in 94% (94/100) of the videos (216/367, 58.9% of coded postural states vs 75/367, 20.4% standing), and figures predominantly held a downward gaze (93/100, 93% of the videos)—which in recovery videos often shifted upward. App videos showed greater physical mobility (33/50, 66% containing standing) compared to API videos (6/50, 12%). Gender differed by modality: app outputs skewed male (34/50, 68%), and API outputs skewed female (33/56, 59%).

#### Recovery Transition Patterns

**Figure 6.**
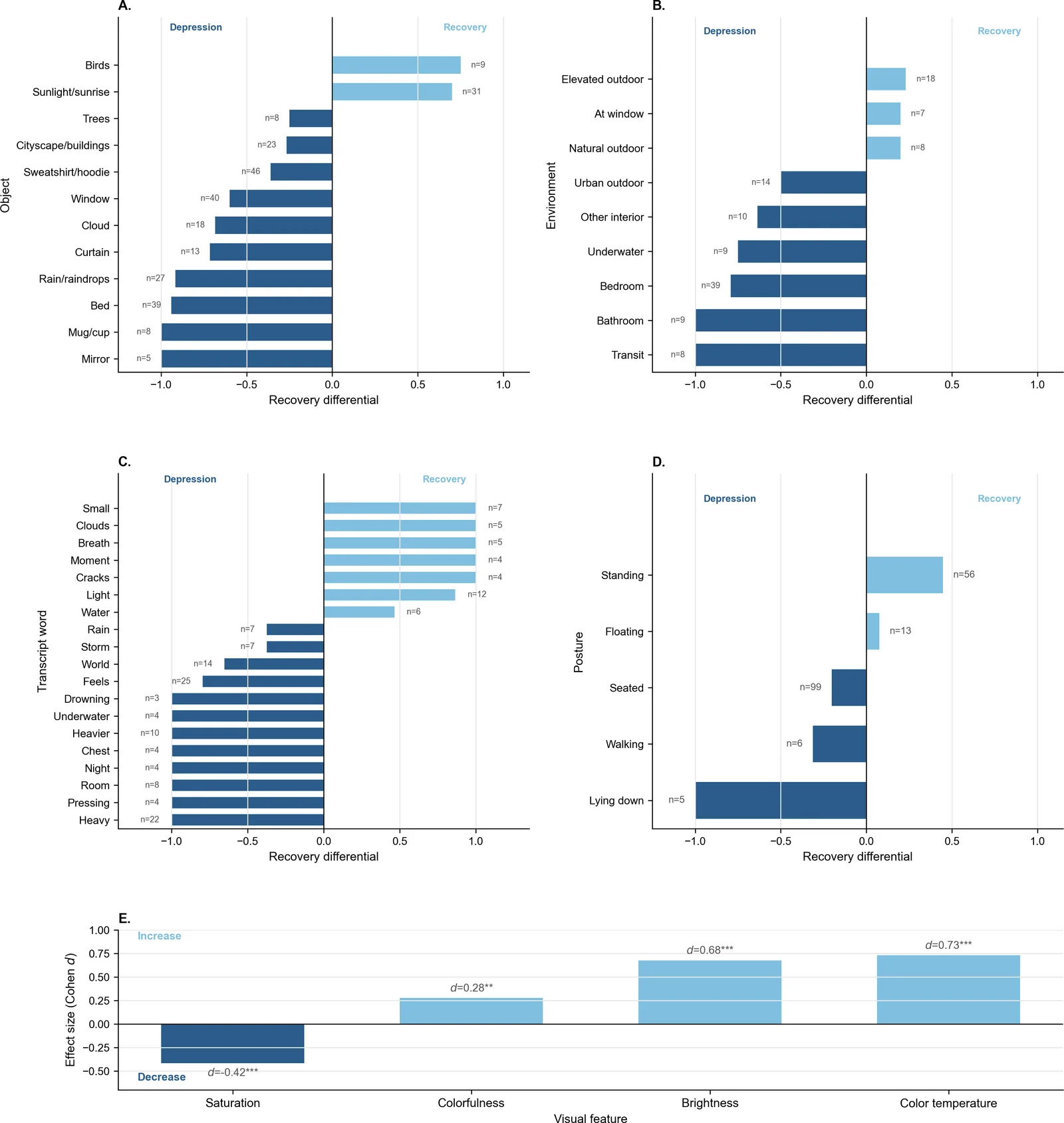
Recovery-linked content shifts in videos with recovery arcs (n=46). (A) Objects, (B) environments, (C) transcript words, and (D) posture codes, plotted as recovery differentials computed as (after-before) and/or (after+before); positive values (light blue) indicate elements appearing predominantly after the coded recovery time stamp, and negative values (dark blue) before it. Bar labels give the number of videos containing each element (A, B, and D) or total word instances (C). Objects and environments required n≥5; objects and transcript words were selected a priori for thematic relevance. (E) Change in visual features after vs before the recovery time stamp, expressed as Cohen *d*. *P*<.05; *P*<.01; *P*<.001.

To examine whether recovery patterns extended beyond coded objects and environments, we analyzed transcript words and quantitative visual features before vs after coded recovery time stamps (46/100, 46% of the videos had recovery arcs; Figure 6).

Analysis of environment appearance relative to recovery shifts (46/100, 46% of the videos) revealed systematic patterns. Environments appearing predominantly before recovery shifts included the bedroom (26/46, 56.5% of the videos before vs 3/46, 6.5% after), the bathroom (7/46, 15.2% before vs 0% after), and the urban outdoors (12/46, 26.1% before vs 4/46, 8.7% after). Elevated outdoor settings (rooftops, hilltops, and bridges) showed the opposite pattern (5/46, 10.9% of the videos before vs 8/46, 17.4% after), appearing predominantly after recovery shifts. Objects followed parallel trajectories. Beds (32/46, 69.6% before vs 1/46, 2.2% after), rain (23/46, 50% before vs 1/46, 2.2% after), windows (32/46, 69.6% before vs 8/46, 17.4% after), and curtains (12/46, 26.1% before vs 2/46, 4.3% after) clustered in depressive phases, whereas sunlight and sunrise appeared predominantly after recovery shifts (3/46, 6.5% before vs 17/46, 37.0% after), as did birds (1/46, 2.2% before vs 7/46, 15.2% after). In terms of figure posture, gaze shifted from downward to upward in 67% (30/45) of recovery videos compared to 17% (9/54) of nonrecovery videos.

Transcript vocabulary reinforced these patterns. Words appearing predominantly before recovery time stamps included “heavy” (n=22), “feels” (n=25), “heavier” (n=10), “room” (n=8), “world” (n=14), “rain” (n=7), and “storm” (n=7). Words appearing predominantly after recovery time stamps included “light” (n=12), “small” (n=7), “clouds” (n=5), “breath” (n=5), “cracks” (n=4), and “moment” (n=4). Depression-phase vocabulary clustered around weight and pressure metaphors (“heavy,” “heavier,” “pressing,” and “chest”) and water and confinement imagery (“underwater,” “drowning,” and “room”), whereas recovery-phase vocabulary emphasized light, smallness, and atmospheric imagery.

Quantitative visual features showed corresponding patterns. Comparing mean values before vs after recovery time stamps, brightness increased by 26.6% (Cohen *d*=0.68; *P*<.001), saturation decreased by 13.2% (Cohen *d*=−0.42; *P*<.001), and colorfulness increased by 10.5% (Cohen *d*=0.28; *P*=.002). Motion showed no significant change (*P*=.93).

## Discussion

### Principal Findings

Our findings indicate that depictions of depression generated by the Sora 2 app depend on more than the underlying model. How that model is embedded and circulated in the product or app has a direct impact on the content, aesthetics, and narrative preferences present in outputs—in ways that diverge from base model behaviors. When prompted with the isolated diagnostic term “Depression,” the consumer-facing Sora app overwhelmingly produced recovery-oriented narratives, whereas the same model accessed through the API yielded largely static, unresolved scenes. In total, 78% (39/50) of app-generated videos contained a discernible shift toward hope or relief compared with only 14% (7/50) of API outputs.

The contrast between product or app and API outputs indicates that product layer mediation shapes how depression is represented. The Sora app positions video generation within a social feed governed by community guidelines that prohibit content perceived as promoting depression or depicting self-harm [8]. Whether this recovery shift bias stems from these explicit content safeguards or emerges indirectly from platform dynamics that favor narrative progression and engagement cannot be determined from our analysis. Regardless of mechanism, our analysis suggests that the Sora 2 product layer introduces strong narrative constraints. Within this environment, outputs are disproportionately oriented toward recovery as a safe and legible end point. Clinically, this suggests that model outputs can be steered toward specific behaviors and that clinical teams can play a role in improving AI video tools at the product level.

Our results are consistent with broader concerns that generative AI systems may favor culturally overrepresented narrative end points (eg, happy endings, resolved arcs, and completed transformations) when responding to ambiguous prompts rather than sustaining unresolved intermediate states. Writers working with language models have described this tendency toward narrative overfitting [39], noting in particular a bias toward positive narrative endings [40]. In video generation, this bias manifests as a rapid exit from stasis into motion, light, elevation, and relief. This pattern may stem from training objectives, or it may encode the dominance of the “restitution narrative” in human-generated content: the culturally preferred illness storyline in which sickness is a temporary interruption resolved through recovery [41]. This dynamic was not absent in the API’s outputs, but it was substantially amplified when the model operated within the Sora 2 app, which is effectively a social distribution pipeline optimized for engagement, shareability, and moderation.

Importantly, the visual and narrative grammars used in these videos are not novel. The withdrawn figure in a dim bedroom, the rain-streaked window, and the gray hoodie all echo iconographies of melancholia traceable from Renaissance allegory and Enlightenment-era psychiatric imaging to modern media depictions, consistent with patterns observed in text-to-image models [23]. The visual grammar also aligns with existing research on depression metaphors that identify darkness, descent, weight, and containment as near-universal conceptual frameworks [42-44]. Our findings seem to confirm these patterns’ persistence in AI video models. The dominance of solitary, seated figures; the clustering of beds, rain, and windows in depressive phases; and the systematic reversal toward light, birds, and elevation during recovery all make use of established symbols and motifs from media. Our findings suggest that Sora 2 does not invent new ways of depicting depression; instead, it compresses and recombines historical visual conventions and routes them through contemporary platform logics that privilege certain end points and user behaviors over others. Clinicians should be aware that AI-generated content about depression reflects training data and platform design rather than clinical expertise and may need to help patients contextualize the simplified narratives and homogeneous imagery they encounter. For researchers and policymakers, these findings reiterate a methodological imperative: analyses of generative AI must attend to *both* base models and the media forms and distribution systems through which AI model outputs are encountered by public user bases.

This study has several limitations. The analysis was restricted to a single platform (Sora 2) and a single prompt term, limiting generalizability to other models, products, prompt formulations, and psychiatric conditions. Sora 2’s training data and moderation pipeline are proprietary and opaque, so we can observe app-API differences but cannot attribute them to specific mechanisms; we also did not conduct human perception studies, so our claims concern output content rather than effects on viewers. Figure demographics were inferred by coders from synthetic figures and, therefore, carry the bias risks inherent in such perceptual judgments. The disparity in default clip length between access points (10 seconds for the app and 12 seconds for the API) introduces a potential confound. Where possible, we mitigated this by analyzing per-second rates and slopes rather than raw totals, but the difference cannot be fully disentangled from product layer effects. Coders were not blinded to access modality as the generation interfaces differed visibly, which may have introduced expectation effects in coding. Finally, the sample of 50 videos per modality was chosen to establish preliminary baseline observations rather than power specific inferential comparisons; effect sizes should be interpreted accordingly.

### Conclusions

This study provides the first systematic analysis of how the Sora 2 generative AI video model depicts depression, revealing that its outputs are shaped as much by product layer mediation as by underlying model associations. The consumer app’s pronounced recovery bias (with 39/50, 78% of videos progressing toward hope compared to only 7/50, 14% of API outputs) demonstrates that platform constraints actively reshape which mental health narratives reach users. Across both access points, the visual and linguistic grammar proved remarkably consistent with centuries-old iconographic traditions: the solitary figure, the dim interior, the downward gaze, and the metaphors of weight and weather. These findings suggest that Sora 2 predominantly recirculates familiar cultural conventions for depicting depression rather than departing sharply from them. We expect that this pattern may well extend to other generative AI video models built on similar architectures and training data. As AI-generated content becomes increasingly prevalent in the informational environments of people experiencing psychiatric distress, clinicians should understand that these outputs reflect training data and platform constraints rather than clinical knowledge and that they may shape patient expectations about recovery timelines and trajectories. Researchers and policymakers, in turn, should attend to the distinction between model behavior and product behavior when evaluating these systems.

## Supplementary material

10.2196/95682 Multimedia Appendix 1 Extended qualitative methods.

10.2196/95682 Multimedia Appendix 2 Qualitative tagging guide.

10.2196/95682 Multimedia Appendix 3 Harmonization dictionaries.

10.2196/95682 Multimedia Appendix 4 Extended quantitative methods.

10.2196/95682 Multimedia Appendix 5 Interrater reliability results.

## Abbreviations

FDR: false discovery rate
SFV: short-form video
