# Supplementary material for: Depictions of Depression in Generative AI Video Models: Mixed Methods Study of OpenAI’s Sora 2
Source: J Med Internet Res. 2026 Sep 14;28:e95682. doi: 10.2196/95682 (PMC13574309; doi:10.2196/95682)
Supplement: Supplementary file 1 [file jmir-v28-e95682-s001.docx]

**Appendix A: Extended Qualitative Methods**

**Coding procedure.** Each video was reviewed in three passes. The first pass was an orientation viewing without pausing to get an overall sense of narrative arc, mood, and content. The second pass involved detailed coding with pausing and frame-by-frame scrubbing as needed to tag all elements with accurate timestamps. The third pass was a verification review to confirm accuracy, fill gaps, and add notes about uncertainties. Coders were instructed to prioritize precision over interpretation (tagging what was directly observable rather than inferred) and to err toward over-tagging when uncertain.

**Coding dimensions.** We coded six dimensions for each video, each selected based on established findings in the depression representation literature.

*Narrative arc* captured whether the video presented a discernible shift in emotional trajectory and, if so, its direction: recovery (movement toward more positive, hopeful, or lighter presentation) or deterioration (movement toward more negative, darker, or heavier presentation). Shifts could be signaled through changes in lighting, color palette, music, figure posture, voiceover language, or environmental elements. Not all videos contained shifts; some presented a consistent mood throughout. This dimension addresses whether Sora 2 tends to depict depression as a static state, a worsening condition, or something that improves. Visual depictions of depression consistently employ trajectory metaphors, either escape from confinement or progressive entrapment, making directional shifts a key marker of how the condition is visually conceptualized [1].

*Environments* were coded with timestamps to capture all physical settings or spaces depicted in each video. We were interested in what kinds of environments Sora 2 associates with depression; whether predominantly indoor, outdoor, domestic, institutional, natural, urban, or abstract. Coders described environments as specifically as the video allowed (e.g., "small bedroom with drawn curtains," "forest path with fog," "geometric void with floating shapes"), using general categories only when more specific description was not possible. Confinement and containment are among the most frequently documented metaphors for depression, often rendered visually through enclosed or restrictive spaces [1].

*Objects* were inventoried with timestamps to capture what visual symbols and props Sora 2 associates with depression. Categories of interest included furniture and fixtures, weather and atmospheric elements, nature, personal items, domestic objects, and symbolically notable items such as empty chairs, wilting plants, or broken glass. Visual symbols of depression draw on embodied metaphor systems linking the condition to darkness, descent, weight, and fragility [1].

*Figure demographics* captured information about human figures depicted in the videos, including apparent gender presentation, race/ethnicity, age (coded by decade), and apparent socioeconomic status (inferred from clothing, grooming, and setting context). This dimension may help reveal whether the model reproduces stereotypical representations of who experiences depression, though as discussed in Appendix E, reliability for some demographic variables proved insufficient for substantive analysis. Figures were tagged even when only partially visible (hands, silhouette, back of head), with visibility limitations noted. Voiceover narration without a visible speaker was treated as a figure, with demographic fields marked "Unclear" when not identifiable from voice alone. Stock imagery of depression disproportionately features young women and carries a more positive emotional tone when depicting men, patterns that may or may not resurface in AI generated videos [2].

*Figure states* captured how figures appeared and behaved over time, including posture (lying down, seated, standing, walking), facial expression, and whether the figure appeared alone in the frame. Because these attributes can change during a figure's appearance (e.g., a figure who begins lying in bed looking sad and ends standing by a window looking neutral) this dimension allowed multiple timestamped entries per figure to capture transitions. Facial expressions were coded using standardized categories: sad, happy/smiling, angry, anxious/worried, neutral/flat, tearful, pensive, tired/exhausted, distressed, unclear, or not visible. Slumped posture and social isolation are characteristic of both clinical presentation and media cliché; coding these features allows assessment of whether generated imagery reproduces narrow visual conventions [3].

*Overall notes* captured general observations, AI artifacts (visual glitches, morphing, distortions), and uncertainties requiring discussion. The full codebook with detailed definitions and edge case handling appears in Appendix B.

**Reliability.** Two authors (MF and ZZ) independently coded the full dataset using the structured codebook. One author's coding (MF) was designated as the primary dataset for analysis; the second author's coding (ZZ) served as inter-rater reliability verification.

Raw codes were harmonized prior to reliability analysis to reconcile minor vocabulary differences between coders. Environment descriptions were mapped to 11 standardized categories (Bedroom, Bathroom, Kitchen/Dining, Other Interior, At Window, Transit, Urban Outdoor, Natural Outdoor, Elevated Outdoor, Underwater, Abstract). Object descriptions were harmonized to standardized forms (e.g., "sweatshirt," "hoodie," "sweater" → "sweatshirt/hoodie"); reliability was calculated at this harmonized object level. Facial expressions were collapsed into three affect categories: Positive (happy, peaceful, hopeful), Subdued/Low (tired, neutral, pensive), and Negative (sad, tearful, worried, distressed, angry). Posture was harmonized to six categories (Seated, Standing, Lying down, Walking, Floating/Swimming, Kneeling/Crouching). Minor variations in demographic coding (e.g., "Medium" vs. "Middle" for SES) were reconciled during harmonization. The full harmonization dictionaries appear in Appendix C.

Cohen's kappa (κ) was calculated for presence/absence of categorical elements within each video, including narrative direction, environment categories, harmonized objects, figure demographics, and figure states. For dimensions involving temporal segments (environments, objects, figure states), we also calculated intersection-over-union (IoU) at one-second resolution to assess agreement on when elements appeared. Coders frequently noted gaze direction in free-text annotations; agreement on gaze was assessed for videos where both coders documented directional looking behavior. Given the scale of the dataset, item-by-item consensus resolution was not feasible; reliability metrics verified that the coding scheme could be applied consistently across coders.

**References**

1. Forceville C, Paling S. The metaphorical representation of depression in short, wordless animation films. Visual communication. 2021 Feb;20(1):100-20.
2. Brassine J, Van den Eynde J, Hubble TR, Toelen J. Digital images of pediatric mental disorders do not accurately represent the conditions. Heliyon. 2020 Sep 1;6(9).
3. Dehcheshmeh TF, Majelan AS, Maleki B. Correlation between depression and posture (A systematic review). Current Psychology. 2024 Sep;43(33):27251-61.
