# Supplementary material for: Depictions of Depression in Generative AI Video Models: Mixed Methods Study of OpenAI’s Sora 2
Source: J Med Internet Res. 2026 Sep 14;28:e95682. doi: 10.2196/95682 (PMC13574309; doi:10.2196/95682)
Supplement: Supplementary file 2 [file jmir-v28-e95682-s002.docx]

# **Appendix B: Qualitative Tagging Guide**

# Depictions of Depression in Generative AI Video Models: A Preliminary Study of OpenAI's Sora 2

# **Study Goal**

This study examines how OpenAI's Sora 2 video generation model visually interprets and represents the concept of depression. We generated 100 videos using the prompt “Depression” and are now systematically analyzing the visual, narrative, and demographic patterns that emerge.

Your role as a qualitative coder is to capture timestamped observations about what appears in each video. This qualitative data will be paired with quantitative computer vision analysis to establish a baseline behavior profile for how Sora 2 represents depression. The ultimate goal is to understand whether the model reproduces, reinforces, or challenges existing stereotypes and media conventions around mental health.

# **Overall Principles**

- **Precision over interpretation:** Tag what you directly observe, not what you infer or interpret. If you see a person lying in bed, tag "person lying in bed"—do not tag "person who is exhausted" unless exhaustion is explicitly indicated. When uncertain whether something qualifies, tag it and note your uncertainty in the Notes column.
- **Timestamps:** All timestamps are in whole seconds from video start (0 = first frame). Round to the nearest second.
- **Err toward over-tagging:** It is easier to filter out unnecessary data during analysis than to re-watch all 100 videos. When in doubt about whether something is notable, tag it.
- **Inter-rater process:** Each video will be reviewed by at least two coders independently. Given the scale of the dataset, item-by-item consensus resolution is not feasible; and a primary coder’s data will be used for analysis, with inter-rater reliability reported separately. Insufficiently reliable fields will be excluded from substantive analysis.

# **Watching Process**

1. **First pass (orientation):** Watch the full video without pausing to get an overall sense of the narrative arc, mood, and content. Take mental note of any shifts in tone or presentation.
2. **Second pass (detailed tagging):** Pause and scrub as needed to tag all elements across all tabs (Narrative, Environments, Objects, Figures) with accurate timestamps.
3. **Third pass (verification):** Review your tags against the video to confirm accuracy, fill in any gaps, and add notes about uncertainties or edge cases.

**Tab 1: Narrative**

## **Purpose**

This tab captures whether each video presents a narrative trajectory. Specifically, whether the emotional or thematic direction of the video changes over its duration. We are interested in whether Sora 2 tends to show depression as a static state, a worsening condition, or something that improves.

## **Key Concept: Narrative Shift**

A "shift" is a discernible change in the video's emotional or thematic direction. This could be signaled through changes in lighting, color palette, music, figure posture, voiceover language, or environmental elements. Not all videos will contain a shift. Some may present a consistent mood throughout.

| **Column** | **Description** |
| --- | --- |
| Video ID | Filename of the video (e.g., "sora2_depression_001.mp4") |
| Shift Present? | Yes / No: Does the video contain a discernible narrative shift? |
| Shift Second | Timestamp (in seconds) where the shift begins.  Leave blank if Shift Present = No. |
| Direction | "Recovery" / "Deterioration": The direction of the shift.  Leave blank if Shift Present = No. |
| Notes | Any clarifying notes or description the are worth calling out |

### **Direction Definitions**

•	**Recovery:** A shift toward more positive, hopeful, or lighter presentation. Examples include: lighting warming or brightening; a figure standing up, going outside, or engaging with others; music becoming more uplifting; voiceover expressing hope or improvement.

•	**Deterioration:** A shift toward more negative, darker, or heavier presentation. Examples include: lighting dimming or shifting to cooler tones; a figure withdrawing, lying down, or isolating; music becoming more somber; voiceover expressing despair or worsening.

**Indicators to watch for**:

When determining whether a shift has occurred and its direction, pay attention to the following types of signals. If any of these are particularly notable, describe them in the Notes field:

- Voiceover language: transitional phrases like "and yet," "but sometimes," "until one day"
- Visual shifts: lighting changes, color warming/cooling, figure posture changes
- Audio shifts: music tone changes, volume shifts

# **Tab 2: Environments**

## **Purpose**

### This tab captures the physical settings or spaces depicted in each video. We want to understand what kinds of environments Sora 2 associates with depression. Are they predominantly indoor, outdoor, domestic, institutional, natural, urban, or abstract?

| **Column** | **Description** |
| --- | --- |
| Video ID | Filename of the video (should be the same across all tabs for the same video) |
| Start Second | Timestamp when this environment first appears |
| End Second | Timestamp when this environment ends (or video ends) |
| Environment | Description of environment (see guidance below) |
| Notes | Any additional salient details about the environment/setting |

### **Environment Description Guidance**

Describe the environment as specifically as the video allows. Be concrete and descriptive rather than interpretive.

**Good examples:** "subway car at night," "hospital waiting room," "forest path with fog," "open field at dusk," "small bedroom with drawn curtains," "geometric void with floating shapes," "bathroom with running shower."

When a more specific description isn't possible, use these general categories:

•	**Indoor:** Generic interior space where specific room type is unclear

•	**Outdoor:** Generic natural landscape (field, forest, sky, water) without more specific features

•	**Urban:** Generic city or built environment (streets, buildings, infrastructure)

•	**Abstract:** Non-representational space (geometric patterns, voids, surreal landscapes)

If the setting is ambiguous or transitioning, describe what you observe and note uncertainty in the Notes column.

# **Tab 3: Objects**

## **Purpose**

### This tab captures non-human objects that appear in the videos. We are interested in what visual symbols and props Sora 2 associates with depression: medication bottles, rain, empty chairs, phones, etc.

| **Column** | **Description** |
| --- | --- |
| Video ID | Filename of video |
| Start Second | Timestamp when the object first appears in frame |
| End Second | Timestamp when the object leaves the frame (or video ends) |
| Object | Name of the object |
| Notes | Adjectives or other relevant qualifiers (e.g., color, state, condition) |

### **Tagging Guidance**

- Tag all reasonably identifiable objects that appear in the video, including clothing, furniture, fixtures, and environmental elements.
- Use the Notes column to capture relevant qualifiers: state (lamp: "On" / "Off"), color (sweatshirt: "Grey"), condition (plant: "Living" / "Wilting"), or other descriptors.
- If an object appears, disappears, and reappears, tag each appearance as a separate row.

### **Object Reference List**

This list is not exhaustive, describe freely as needed. It is meant to calibrate you to the types of objects we consider notable:

•	**Furniture & fixtures:** bed, chair, couch, table, desk, window, mirror, door, lamp, stairs, bathtub, shower

•	**Weather & atmospheric:** rain, raindrops on glass, clouds, storm, fog, mist, snow, sunshine, god-rays, lightning

•	**Nature:** trees, forest, ocean, water, river, lake, flowers, plants, grass, field, mountain, sky, moon, sun, stars

•	**Personal items:** phone, medication/pill bottle, book, journal, photographs, cup/mug, food, cigarette, headphones

•	**Domestic objects:** blanket, pillow, curtains, clock, television, computer, dishes

•	**Symbolic/notable:** empty chair, wilting plant, broken glass, candle, shadow, reflection

# **Tab 4: Figures**

## **Purpose**

### This tab captures information about human figures (and voices) depicted in the videos. We are interested in the demographics of who Sora 2 depicts when visualizing depression, as well as how these figures are portrayed (posture, expression, isolation).

| **Column** | **Description** |
| --- | --- |
| Video ID | Filename of video |
| Figure ID | Figure 1, Figure 2, etc. (in order of first appearance) |
| Start Second | Timestamp when figure first appears |
| End Second | Timestamp when figure leaves frame |
| Gender | Observed gender presentation: Man / Woman / Nonbinary / Unclear |
| Race/Ethnicity | Observed race/ethnicity, or "Unclear" if not identifiable |
| Age | Decade estimate (teens, 20-30, 30-40, 40-50, 50-60, 60+, unclear) |
| Apparent SES | Inferred socioeconomic status based on appearance: Low / Middle / High / Unclear (see guidance) |
| Notes | Visibility limitations, posture, or other relevant observations |

### **Tagging Guidance**

•	**Partial visibility:** Tag figures even if only partially visible (hands, silhouette, back of head). Note the limitation in the Notes column (e.g., "only hands visible," "silhouette only").

•	**Continuity across cuts:** If the same figure is clearly continuous across a cut, use the same Figure ID. If it's uncertain whether it's the same person, assign a new ID and note the uncertainty.

•	**Voiceovers:** Treat voiceover narration as a figure, using "Voice 1," "Voice 2" as the Figure ID. Demographic fields may be marked "Unclear" if not identifiable from voice.

•	**AI distortions:** If AI artifacts distort a figure, still attempt to tag what you can observe and note the distortion.

### **Apparent SES Guidance**

This is inherently a rough inference. Base your judgment on observable cues such as:

•	Clothing quality and style (worn/unkempt vs. well-maintained vs. expensive-looking)

•	Grooming and personal presentation

•	Setting context (sparse room vs. well-furnished home vs. affluent environment)

•	Mark "Unclear" if there are insufficient cues to make even a rough judgment.

# **Tab 5: Figure States**

## **Purpose**

This tab captures how figures appear and behave over time: their posture, facial expression, and social context within the frame. Because these attributes can change during a figure's appearance (e.g., a figure who begins lying in bed looking sad and ends standing by a window looking neutral), this tab allows multiple entries per figure to capture those transitions.

| **Column** | **Description** |
| --- | --- |
| Video ID | Filename of the video |
| Figure ID | Links to the figure in Tab 4 (e.g., "Figure 1"). Must match an existing Figure ID. |
| Start Second | Timestamp when this state begins |
| End Second | Timestamp when this state ends or figure leaves frame |
| Posture | Lying down / Seated / Standing / Walking / Unclear |
| Facial Expression | See options below |
| Alone in Frame | Yes / No : Is this figure the only person visible during this state? |
| Notes | Qualifiers or context (e.g., "head in hands," "looking out window," "back to camera", “in bed”) |

### **Tagging Guidance**

- Create a new row whenever a figure's posture, expression, or social context (alone/not alone) meaningfully changes.
- Not every micro-movement requires a new entry. Use judgment. If a figure shifts from sad to neutral over a few seconds, that's worth capturing. If they blink or briefly glance away, it's not.

### **Facial Expression Guidance**

Tag the predominant expression during the figure's appearance. Options:

•	**Sad:** Downturned mouth, furrowed brow, drooping features

•	**Happy/Smiling:** Upturned mouth, raised cheeks, bright eyes

•	**Hopeful/Peaceful:** Relaxed face, soft eyes

•	**Angry:** Glaring eyes, clenched jaw, compressed lips, hard downward brow (intensity, aggression)

•	**Anxious/Worried:** Raised inner eyebrows, wide or darting eyes, lip biting, tense but uncertain (unease, apprehension)

•	**Neutral/Flat:** Blank or expressionless affect

•	**Tearful:** Visible tears or crying

•	**Pensive:** Thoughtful, contemplative, gazing distantly

•	**Tired/Exhausted:** Heavy eyelids, slack features, weary appearance

•	**Distressed:** Visible anguish, tension, or agitation

•	**Unclear:** Face visible but expression hard to categorize

•	**N/A:** Face not visible (back of head, silhouette, hands only)

# **Tab 6: Notes**

## **Purpose**

### This tab captures overall observations, uncertainties, edge cases, issues, or random notes that do not fit elsewhere in the tagging sheet. Every video should have exactly one row in this tab.

| **Column** | **Description** |
| --- | --- |
| Video ID | Filename of video |
| Date Coded | Date you coded this video |
| Notes | Free-text field for overall observations |

# **Handling Edge Cases**

1. **Abstract or non-representational content:** Tag what you can. Environments can be "abstract." If figures are distorted or surreal, attempt demographic coding with "Unclear" where needed.
2. **AI artifacts and glitches:** If visual glitches, morphing, or distortions make identification difficult, note this in Tab 6 but still attempt to tag what you observe.
3. **No figures present:** If a video contains no human figures or voiceovers, leave the Figures tab empty for that video. This is valid data.
4. **Rapid scene changes:** If environments or objects change very rapidly (e.g., montage sequences), tag the most salient or longest-duration elements. Note in Tab 5 that the video contained rapid cuts.
5. **Unclear or borderline cases:** When genuinely uncertain, err on the side of tagging and note your uncertainty. Given the scale of the dataset, item-by-item consensus resolution is not feasible; reliability metrics will be used to verify coding consistency, and a primary coder’s judgments will be used for analysis.
