# Supplementary material for: Depictions of Depression in Generative AI Video Models: Mixed Methods Study of OpenAI’s Sora 2
Source: J Med Internet Res. 2026 Sep 14;28:e95682. doi: 10.2196/95682 (PMC13574309; doi:10.2196/95682)
Supplement: Supplementary file 3 [file jmir-v28-e95682-s003.docx]

# Appendix C: Harmonization Dictionaries

To reconcile minor vocabulary differences between coders and enable systematic analysis, raw codes were mapped to standardized categories. All mappings used case-insensitive substring matching unless otherwise noted.

## **Environment Harmonization**

| **Standardized Category** | **Raw Code Patterns** |
| --- | --- |
| Bedroom | "bedroom", "in bed" |
| Bathroom | "bathroom", "bathtub", "in bathtub" |
| Kitchen/Dining | "kitchen", "dining", "cafe" |
| Other Interior | "desk", "living room", "hallway", "basement", "empty room", "elevator" |
| At Window | "window", "at window", "next to window", "in front of window" |
| Transit | "bus", "subway", "train", "car", "transit" |
| Urban Outdoor | "street", "city", "urban", "underpass", "parking" |
| Natural Outdoor | "park", "field", "meadow", "river", "lake", "lagoon", "pond", "wheat", "flower", "garden", "ice flat", "outside" |
| Elevated Outdoor | "roof", "rooftop", "hilltop", "mountain", "balcony", "top of", "bridge" |
| Underwater | "underwater", "under water", "submerged", "in water" |
| Abstract | "space", "cloud", "abstract", "galaxy", "sky", "dome" |

*Notes: "underwater bedroom" mapped to Underwater. Close-up shots carried forward the previous environment.*

## **Object Harmonization**

| **Standardized Object** | **Raw Code Patterns** |
| --- | --- |
| sweatshirt/hoodie | "sweatshirt", "hoodie", "hoddie", "sweater", "long-sleeve", "long sleeve", "t-shirt", "shirt" |
| jacket/coat | "jacket", "coat", "rainjacket", "raincoat", "blazer" |
| bed | "bed" (excluding "bedroom") |
| couch/sofa | "couch", "sofa" |
| end table | "end table", "nightstand" |
| table/desk | "table", "desk" |
| rain | "rain", "heavy rain" |
| raindrops | "raindrop", "rain on" |
| cloud | "cloud" |
| sunrise/sunset | "sunrise", "sunset" |
| sunlight/god rays | "god ray", "god-ray", "sunlight" |
| standing water/puddles | "standing water", "water on", "puddle" |
| bathtub | "bathtub" |
| bubbles | "bubble" |
| plant/flowers | "plant", "flower", "sunflower" |
| trees | "Tree", “trees” |
| birds | "bird" |
| phone | "phone" |
| mug/cup | "mug", "cup" |
| lamp | "lamp" |
| window | "window", "windows" |
| curtain | "Curtain", “curtains” |
| tear | "tear" |
| mirror | "mirror" |
| hands | "hands", "hand" |
| cityscape/buildings | "cityscape", "building", "skyline" |
| cars | "car", "cars" |
| picture/poster | "picture", "poster", "photo", "polaroid" |
| bench | "bench" |

*Note: Objects not matching any pattern were retained as-is.*

## **Figure State Harmonization**

### Posture

| **Standardized Category** | **Raw Code Patterns** |
| --- | --- |
| Seated | "seated", "sitting", "seted" |
| Standing | "standing", "standng", "stading" |
| Lying down | "lying" |
| Walking | "walking" |
| Floating/Swimming | "floating", "swimming" |
| Kneeling/Crouching | "kneeling", "crouching", "squatting", "crawling" |

### Facial Expression

| **Affect Category** | **Raw Codes** |
| --- | --- |
| Positive | "happy", "hopeful", "peaceful", "surprised" |
| Subdued/Low | "tired", "neutral", "pensive", "flat", "focused", "sleeping" |
| Negative | "sad", "tearful", "crying", "worried", "distressed", "anxious", "angry", "desperate", "dejected" |

### Alone in Frame

| **Standardized** | **Raw Codes** |
| --- | --- |
| Yes | "yes" |
| No | "no" |

## **Figure Demographic Harmonization**

### Apparent SES

| **Standardized** | **Raw Codes** |
| --- | --- |
| High | "high" |
| Medium | "medium", "middle" |
| Low | "low" |

### Race/Ethnicity

| **Standardized** | **Raw Codes** |
| --- | --- |
| Black | "black" |
| White | "white" |
| Latino/Hispanic | "hispanic", "latino", "latina" |
| Asian | "asian" |
| Arab/Middle Eastern | "arab", "middle eastern" |
| Indian | "indian" |

### Gender

| **Standardized** | **Raw Codes** |
| --- | --- |
| Male | "male" |
| Female | "female" |

### Age

| **Standardized** | **Raw Codes** |
| --- | --- |
| 10-20 | "teens", "10-20" |
| 20-30 | "20-30" |
| 30-40 | "30-40" |
| 40-50 | "40-50" |
| 50-60 | "50-60" |

### Gaze Direction Extraction (from Free-Text Notes)

| **Gaze Direction** | **Note Patterns** |
| --- | --- |
| Down | "looking down", "looking at hands", "looking at the ground", "head in hands", "face in hands", "eyes closed", "looking at feet", "looking at lap" |
| Up | "looking up", "looking at the sky", "looking at sunset", "looking at sunrise", "looking at the cloud", "looking at clouds", "looking at lights", "looking to the sky" |
| Out/Away | "looking out", "looking outside", "looking at window", "out of window", "out window", "looking far", "looking afar", "looking into distance", "looking away", "looking around", "in front of window", "checking outside" |
