# Supplementary material for: Depictions of Depression in Generative AI Video Models: Mixed Methods Study of OpenAI’s Sora 2
Source: J Med Internet Res. 2026 Sep 14;28:e95682. doi: 10.2196/95682 (PMC13574309; doi:10.2196/95682)
Supplement: Supplementary file 4 [file jmir-v28-e95682-s004.docx]

**Appendix D: Extended Quantitative Methods**

All feature extraction was implemented in Python using a custom analysis pipeline. The analysis code and video dataset analyzed in this study are available from the corresponding author upon reasonable request.

**D.1 Visual Aesthetics**

We extracted frames at one-second intervals, yielding 11 frames for 10-second app videos and 13 frames for 12-second API videos.

**Color and Light Measures**

Each measure was selected based on established connections to mood and emotion in prior research. Brightness, measured as perceptual luminance, has established connections to mood representation [1]. Saturation captures color intensity; reduced saturation is associated with diminished emotional arousal [2]. Colorfulness was computed using the Hasler-Süsstrunk metric [3], which correlates with human perception of chromatic vividness. Color temperature indexes the warm-cool balance; warm tones are associated with comfort and social connection, while cool tones connote isolation [4].

| **Measure** | **Computation** |
| --- | --- |
| Brightness | <https://www.codecogs.com/eqnedit.php?latex=%5Ctext%7BBrightness%7D%20%3D%200.299R%20%2B%200.587G%20%2B%200.114B#0> |
| Saturation | S channel of the HSV color space, computed as the difference between maximum and minimum RGB values divided by the maximum |
| Colorfulness | <https://www.codecogs.com/eqnedit.php?latex=%5Ctext%7BColorfulness%7D%20%3D%20%5Csqrt%7B%5Csigma_%7Brg%7D%5E2%20%2B%20%5Csigma_%7Byb%7D%5E2%7D%20%2B%200.3%20%5Ctimes%20%5Csqrt%7B%5Cmu_%7Brg%7D%5E2%20%2B%20%5Cmu_%7Byb%7D%5E2%7D#0>  where <https://www.codecogs.com/eqnedit.php?latex=rg#0> and <https://www.codecogs.com/eqnedit.php?latex=yb#0> represent opponent color channels <https://www.codecogs.com/eqnedit.php?latex=(rg%20%3D%20R%20-%20G%3B%20yb%20%3D%200.5(R%20%2B%20G)%20-%20B)#0>, and <https://www.codecogs.com/eqnedit.php?latex=%CF%83#0> and <https://www.codecogs.com/eqnedit.php?latex=%CE%BC#0> denote standard deviation and mean respectively. |
| Color temperature | Mean <https://www.codecogs.com/eqnedit.php?latex=(R-B)%20%2B%200.3%20%5C%5Ctimes%20(R-G)#0> channel difference, with positive values indicating warmer colors and negative values indicating cooler colors. |
| Edge Density | Proportion of Canny edge pixels to total pixels    <https://www.codecogs.com/eqnedit.php?latex=%5Ctext%7BEdge%20Density%7D%20%3D%20%5Cfrac%7B%5Csum%20%5Cmathbf%7B1%7D%5B%5Ctext%7Bedge%7D(x%2Cy)%20%3E%200%5D%7D%7BW%20%5Ctimes%20H%7D#0>   where <https://www.codecogs.com/eqnedit.php?latex=W#0> and <https://www.codecogs.com/eqnedit.php?latex=H#0> are frame dimensions. Adaptive thresholds were set at 0.7× and 1.3× the median pixel intensity to account for varying image brightness. |

**Texture Measures**

We also extracted texture features using Gray-Level Co-occurrence Matrices [5], which provide insight into environmental qualities. Smooth textures may suggest sterile spaces, while complex textures indicate detailed or chaotic environments. We computed the GLCM at distance=1 pixel across four angles (0°, 45°, 90°, 135°) and averaged the resulting features.

| **Measure** | **Formula** | **Interpretation** |
| --- | --- | --- |
| Contrast | <https://www.codecogs.com/eqnedit.php?latex=%5Csum_%7Bi%2Cj%7D%20(i-j)%5E2%20%5Ccdot%20P(i%2Cj)#0> | Local intensity variation |
| Dissimilarity | <https://www.codecogs.com/eqnedit.php?latex=%5Csum_%7Bi%2Cj%7D%20%7Ci-j%7C%20%5Ccdot%20P(i%2Cj)#0> | Average difference between neighboring pixels |
| Homogeneity | <https://www.codecogs.com/eqnedit.php?latex=%5Csum_%7Bi%2Cj%7D%20%5Cfrac%7BP(i%2Cj)%7D%7B1%20%2B%20(i-j)%5E2%7D#0> | Closeness of distribution to diagonal |
| Energy | $\sqrt{\sum_{i,j} P\left( i,j \right)^{2}}$ | Uniformity of gray level distribution |
| Correlation | <https://www.codecogs.com/eqnedit.php?latex=%5Csum_%7Bi%2Cj%7D%20%5Cfrac%7B(i%20-%20%5Cmu_i)(j%20-%20%5Cmu_j)%20%5Ccdot%20P(i%2Cj)%7D%7B%5Csigma_i%20%5Csigma_j%7D#0> | Linear dependency of gray levels |
| Entropy | <https://www.codecogs.com/eqnedit.php?latex=-%5Csum_%7Bi%2Cj%7D%20P(i%2Cj)%20%5Ccdot%20%5Clog%5C_2(P(i%2Cj))#0> | Textural randomness or complexity |

where<https://www.codecogs.com/eqnedit.php?latex=P(i%2Cj)#0> is the normalized co-occurrence matrix.

**D.2 Audio Properties**

Audio was extracted from video files and analyzed using the librosa library [6]. These acoustic features have established links to depressive affect: depressed speech is characterized by reduced volume, lower pitch, reduced pitch variability, and lower spectral centroid compared to non-depressed speech [7-8].

| **Measure** | **Formula** | **Interpretation** |
| --- | --- | --- |
| Volume | Root-mean-square (RMS) energy with frame length of 2048 samples and hop length of 512 samples | Acoustic intensity |
| Pitch | Fundamental frequency (F0) extracted using the pYIN algorithm [9], which combines a probabilistic YIN estimator with a hidden Markov model. Mean F0 computed across all voiced frames | Vocal register |
| Spectral centroid | <https://www.codecogs.com/eqnedit.php?latex=%5Ctext%7BCentroid%7D%20%3D%20%5Cfrac%7B%5Csum_%7Bk%7D%20f_k%20%5Ccdot%20M_k%7D%7B%5Csum_%7Bk%7D%20M_k%7D#0>  where <https://www.codecogs.com/eqnedit.php?latex=f_k#0> is the frequency of bin <https://www.codecogs.com/eqnedit.php?latex=k#0> and <https://www.codecogs.com/eqnedit.php?latex=M_k#0> is the magnitude. | "Center of mass" of the frequency spectrum; higher values indicate brighter, more energetic audio, lower values suggest muffled or subdued soundscapes |

**D.3 Semantic Content**

Spoken audio was transcribed using OpenAI's Whisper model (base) [10], with word-level timestamps extracted for temporal analysis. We computed sentiment scores, custom lexicons, and linguistic markers selected based on prior research linking these features to depression and recovery discourse.

*Sentiment and Speech Measures*

We utilized a VADER [33] computed compound, positive, negative, and neutral sentiment ratings for each transcription. VADER is calibrated for social media text and handles informal language, emoticons, and emphasis markers.

| **Measure** | **Computation** |
| --- | --- |
| Word Count | Total words per video |
| Speech Rate | Words per second of video duration |
| Sentiment | VADER compound, positive, negative, and neutral scores |

*Light/Dark Lexicons*

We developed custom lexicons based on conceptual metaphor theory [11] and research showing that depression discourse frequently employs metaphors of weight, darkness, and drowning, while recovery narratives invoke lightness and release [12]. Using word-level timestamps from Whisper, we tracked when these terms appeared across the video timeline. Ambiguous terms that could appear in neutral contexts were excluded.

| **Lexicon** | **Terms** |
| --- | --- |
| Light (hope/recovery) | hope, hopeful, hoping, relief, relieved, release, released, free, freedom, peace, peaceful, calm, calming, serene, serenity, heal, healing, healed, recover, recovery, recovering, joy, joyful, happy, happiness, smile, smiling, laugh, laughing, laughter, love, loved, loving, comfort, comforting, comfortable, safe, safety, warm, warmth, support, supported, supporting, embrace, embraced |
| Dark (distress) | heavy, heaviness, burden, burdened, weigh, weighing, crush, crushing, crushed, drown, drowning, drowned, suffocate, suffocating, choke, choking, trap, trapped, stuck, confined, cage, caged, alone, lonely, loneliness, isolated, isolation, abandoned, forgotten, empty, emptiness, void, hollow, numb, numbness, pain, painful, hurt, hurting, ache, aching, suffer, suffering, exhaust, exhausted, exhausting, drain, drained, draining, weary, fatigue, despair, despairing, hopeless, hopelessness, helpless, helplessness, worthless, worthlessness, fear, afraid, scared, terrified, anxiety, anxious, panic, dread |

*Linguistic Pattern Measures*

We computed linguistic markers based on findings from computational studies of depression. First-person singular pronoun ratio indexes self-focused attention, which has been consistently linked to depressive rumination [13]. Negation frequency captures negative cognitive framing characteristic of depressive thought patterns [14].

| **Measure** | **Numerator** | **Denominator** |
| --- | --- | --- |
| First-person singular pronoun ratio | Count of: I, me, my, mine, myself | Total words |
| Negation ratio | Count of: no, not, never, nothing, none, nobody, nowhere, neither, nor, can't, couldn't, won't, wouldn't, don't, doesn't, didn't, shouldn't, isn't, aren't, wasn't, weren't, haven't, hasn't, hadn't | Total words |
| Lexical diversity (TTR) | Unique words | Total words |

**D.4 Temporal Dynamics**

We computed motion, scene structure, and feature trajectories to analyze whether videos depicted stasis, deterioration, recovery, or cyclical patterns over their duration. Motion magnitude is particularly relevant to depression representation because psychomotor retardation (slowed movement and activity) is a core diagnostic feature of major depression [15].

*Motion Analysis*

Dense optical flow was computed using the Farnebäck algorithm [16] between consecutive frames at native framerate (30 fps). The algorithm estimates motion vectors for every pixel using polynomial expansion. Flow magnitude was computed as<https://www.codecogs.com/eqnedit.php?latex=%5Csqrt%7Bu%5E2%20%2B%20v%5E2%7D#0> where<https://www.codecogs.com/eqnedit.php?latex=u#0> and<https://www.codecogs.com/eqnedit.php?latex=v#0> are horizontal and vertical flow components. Frame-level values were binned into per-second averages for trajectory analysis.

| **Parameter** | **Value** |
| --- | --- |
| Pyramid scale | 0.5 |
| Pyramid levels | 3 |
| Window size | 15 |
| Iterations | 3 |
| Polynomial neighborhood | 5 |
| Polynomial sigma | 1.2 |

*Scene Detection*

Scene cuts were detected by computing histogram correlation between frames sampled at one-second intervals. A cut was registered when correlation dropped below 0.3. Scene cut frequency provides insight into editing rhythm as rapid cuts may signal narrative progression or emotional intensity, while continuous shots suggest sustained states.

*Feature Trajectories*

For each video, per-second values were computed for all features. Linear slope was computed via ordinary least squares to characterize whether features increased, decreased, or remained stable over the video duration

<https://www.codecogs.com/eqnedit.php?latex=%5Ctext%7Bslope%7D%20%3D%20%5Cfrac%7B%5Csum_%7Bt%7D(t%20-%20%5Cbar%7Bt%7D)(x_t%20-%20%5Cbar%7Bx%7D)%7D%7B%5Csum_%7Bt%7D(t%20-%20%5Cbar%7Bt%7D)%5E2%7D#0>

**D.5 Statistical Formulas**

We used standard inferential statistics to compare features between App and API outputs, with corrections for multiple comparisons across all features tested.

*Group Comparisons*

Welch's t-test [17] was used for all between-group comparisons because it does not assume equal variances across groups; an appropriate choice given the substantial differences in output characteristics between modalities.

<https://www.codecogs.com/eqnedit.php?latex=t%20%3D%20%5Cfrac%7B%5Cbar%7BX%7D_1%20-%20%5Cbar%7BX%7D_2%7D%7B%5Csqrt%7B%5Cfrac%7Bs_1%5E2%7D%7Bn_1%7D%20%2B%20%5Cfrac%7Bs_2%5E2%7D%7Bn_2%7D%7D%7D#0>

*Effect Sizes*

Cohen's d [18] was used to quantify the magnitude of differences between App and API outputs, providing a standardized measure independent of sample size.

<https://www.codecogs.com/eqnedit.php?latex=d%20%3D%20%5Cfrac%7B%5Cbar%7BX%7D_1%20-%20%5Cbar%7BX%7D2%7D%7Bs%7Bpooled%7D%7D#0>

where<https://www.codecogs.com/eqnedit.php?latex=s_%7Bpooled%7D%20%3D%20%5Csqrt%7B%5Cfrac%7B(n_1-1)s_1%5E2%20%2B%20(n_2-1)s_2%5E2%7D%7Bn_1%2Bn_2-2%7D%7D#0>

Effect sizes were interpreted using conventional thresholds: |d| = 0.2 (small), 0.5 (medium), 0.8 (large).

*Multiple Comparison Correction*

To control the false discovery rate when conducting multiple comparisons, p-values were adjusted using the Benjamini-Hochberg procedure [19] which provides a principled balance between Type I error control and statistical power:

The procedure operates as follows:

1. Rank all m p-values from smallest to largest:<https://www.codecogs.com/eqnedit.php?latex=p_%7B(1)%7D%20%5Cleq%20p_%7B(2)%7D%20%5Cleq%20%5Cldots%20%5Cleq%20p_%7B(m)%7D#0>
2. For each ranked p-value, compute the critical value:<https://www.codecogs.com/eqnedit.php?latex=%5Cfrac%7Bk%7D%7Bm%7D%20%5Ctimes%20%5Calpha#0>
3. Find the largest k such that<https://www.codecogs.com/eqnedit.php?latex=p_%7B(k)%7D%20%5Cleq%20%5Cfrac%7Bk%7D%7Bm%7D%20%5Ctimes%20%5Calpha#0>
4. Reject all null hypotheses for tests with rank<https://www.codecogs.com/eqnedit.php?latex=%5Cleq%20k#0>

Adjusted p-values (q-values) were computed as:

<https://www.codecogs.com/eqnedit.php?latex=q_%7B(k)%7D%20%3D%20%5Cmin%5Cleft(%5Cfrac%7Bm%20%5Ccdot%20p_%7B(k)%7D%7D%7Bk%7D%2C%201%5Cright)#0>

The raw transformed values were then made monotone by processing from rank m down to rank 1, replacing each q(k) with the minimum of its own value and the q-value at rank k+1, ensuring that adjusted p-values respect the ordering of the original p-values. We used α = 0.05 for all analyses. Where relevant throughout the results, we report FDR-corrected q-values rather than raw p-values.

**D.6 Qualitative Interrater Reliability Methods**

Two authors (MF and ZZ) independently coded the complete dataset of 100 videos using the structured codebook, producing parallel sets of timestamped codes across all dimensions. One author's coding (MF) was designated as the primary dataset for analysis. The second author's independent coding (ZZ) was used to calculate inter-rater reliability.

*Cohen's Kappa*

Agreement on categorical dimensions was assessed using Cohen's kappa (κ) [20]:

<https://www.codecogs.com/eqnedit.php?latex=%5Ckappa%20%3D%20%5Cfrac%7Bp_o%20-%20p_e%7D%7B1%20-%20p_e%7D#0>

where<https://www.codecogs.com/eqnedit.php?latex=p_o#0> is the observed proportion of agreement and<https://www.codecogs.com/eqnedit.php?latex=p_e#0> is the proportion of agreement expected by chance:

<https://www.codecogs.com/eqnedit.php?latex=p_e%20%3D%20%5Csum_%7Bk%7D%20p_%7B1k%7D%20%5Ccdot%20p_%7B2k%7D#0>

where<https://www.codecogs.com/eqnedit.php?latex=p_%7B1k%7D#0> and<https://www.codecogs.com/eqnedit.php?latex=p_%7B2k%7D#0> are the proportions of each category assigned by coders 1 and 2, respectively.

Kappa was calculated for:

- Narrative shift presence (Yes/No)
- Narrative direction (Recovery/Deterioration)
- Environment categories (presence/absence per category per video)
- Object categories (presence/absence per harmonized object per video)
- Figure demographics (gender, race/ethnicity, age range, apparent SES)
- Figure states (posture, facial expression, alone in frame)
- Gaze direction (presence/absence of down, up, out/away per video)

Kappa values were interpreted following Landis and Koch [21]:

| **κ Value** | **Interpretation** |
| --- | --- |
| < 0.00 | Poor |
| 0.00–0.20 | Slight |
| 0.21–0.40 | Fair |
| 0.41–0.60 | Moderate |
| 0.61–0.80 | Substantial |
| 0.81–1.00 | Almost perfect |

*Temporal Intersection over Union*

For dimensions involving timestamped segments (environments, objects, figure states), kappa captures only whether coders agreed that a category was *present* in a video, not whether they agreed on *when* it appeared. To assess temporal agreement, we computed Intersection over Union (IoU) at one-second resolution:

<https://www.codecogs.com/eqnedit.php?latex=%5Ctext%7BIoU%7D%20%3D%20%5Cfrac%7B%7CA%20%5Ccap%20B%7C%7D%7B%7CA%20%5Ccup%20B%7C%7D#0>

where A and B are binary temporal masks indicating the seconds during which each coder marked a category as present. For each video, we created binary vectors with one element per second of video duration (11 elements for 10-second app videos, 13 elements for 12-second API videos) for each coder's segment annotations, then computed the intersection and union across these vectors.

IoU ranges from 0 (no temporal overlap) to 1 (perfect temporal agreement). We computed IoU only for categories where both coders agreed the element was present, providing a measure of boundary precision conditional on presence agreement.

*Timestamp Precision*

For narrative shift timestamps (a single moment rather than a segment), we computed the mean and median absolute difference between coders' timestamps for videos where both identified a shift:

<https://www.codecogs.com/eqnedit.php?latex=%5Ctext%7BPrecision%7D%20%3D%20%7Ct_%7B%5Ctext%7BMF%7D%7D%20-%20t_%7B%5Ctext%7BZZ%7D%7D%7C#0>

Given the scale of the dataset (thousands of individual codes across six coding dimensions) item-by-item consensus resolution was not feasible. Reliability metrics were used to verify that the coding scheme could be applied consistently across coders, with the primary coder's judgments (MF) used for all analyses.

**D.7 References**

1. Dael N, Perseguers MN, Marchand C, Antonietti JP, Mohr C. Put on that colour, it fits your emotion: Colour appropriateness as a function of expressed emotion. Quarterly Journal of Experimental Psychology. 2016 Aug;69(8):1619-30.
2. Wilms L, Oberfeld D. Color and emotion: effects of hue, saturation, and brightness. Psychological research. 2018 Sep;82(5):896-914.
3. Hasler D, Suesstrunk SE. Measuring colorfulness in natural images. InHuman vision and electronic imaging VIII 2003 Jun 17 (Vol. 5007, pp. 87-95). SPIE.
4. Valdez P, Mehrabian A. Effects of color on emotions. Journal of experimental psychology: General. 1994 Dec;123(4):394.
5. Haralick RM, Shanmugam K, Dinstein IH. Textural features for image classification. IEEE Transactions on systems, man, and cybernetics. 2007 Nov 12(6):610-21.
6. Mcfee B, Raffel C, Liang D, Ellis DP, Mcvicar M, Battenberg E, Nieto O. Librosa–audio processing Python library. Inproceedings of the 14th python in science conference 2015 (pp. 18-25).
7. Martínez-Nicolás I, Criado D, Gordillo F, Martínez-Sánchez F, Meilán JJ. Speech analysis for detecting depression in older adults: a systematic review. Frontiers in Psychology. 2025 Dec 12;16:1715538.
8. Mundt JC, Vogel AP, Feltner DE, Lenderking WR. Vocal acoustic biomarkers of depression severity and treatment response. Biological psychiatry. 2012 Oct 1;72(7):580-7.
9. Mauch M, Dixon S. pYIN: A fundamental frequency estimator using probabilistic threshold distributions. In2014 ieee international conference on acoustics, speech and signal processing (icassp) 2014 May 4 (pp. 659-663). IEEE.
10. Radford A, Kim JW, Xu T, et al. Robust speech recognition via large-scale weak supervision [Preprint]. arXiv;2022 Dec 6 [cited 2026 Jan 20]. arXiv:2212.04356. Available from:https://arxiv.org/abs/2212.04356
11. Lakoff G, Johnson M. Metaphors We Live By. Chicago: University of Chicago Press; 1980
12. Malkomsen A, Røssberg JI, Dammen T, Wilberg T, Løvgren A, Ulberg R, Evensen J. Digging down or scratching the surface: how patients use metaphors to describe their experiences of psychotherapy. BMC psychiatry. 2021 Oct 27;21(1):533.
13. Brockmeyer T, Zimmermann J, Kulessa D, Hautzinger M, Bents H, Friederich HC, Herzog W, Backenstrass M. Me, myself, and I: self-referent word use as an indicator of self-focused attention in relation to depression and anxiety. Frontiers in psychology. 2015 Oct 9;6:1564.
14. Amorese T, Cuciniello M, Greco C, Sheveleva O, Cordasco G, Glackin C, McConvey G, Callejas Z, Esposito A. Detecting depression in speech using verbal behavior analysis: a cross-cultural study. Frontiers in Psychology. 2025 May 29;16:1514918.
15. Buyukdura JS, McClintock SM, Croarkin PE. Psychomotor retardation in depression: biological underpinnings, measurement, and treatment. Progress in Neuro-Psychopharmacology and Biological Psychiatry. 2011 Mar 30;35(2):395-409.
16. Farnebäck G. Two-frame motion estimation based on polynomial expansion. InScandinavian conference on Image analysis 2003 Jun 24 (pp. 363-370). Berlin, Heidelberg: Springer Berlin Heidelberg.
17. Welch BL. The generalization of 'student's' problem when several different population variances are involved. Biometrika. 1947;34(1-2):28-35.
18. Cohen J. Statistical power analysis for the behavioral sciences. 2nd ed. Hillsdale (NJ):Lawrence Erlbaum Associates;1988.
19. Benjamini Y, Hochberg Y. Controlling the false discovery rate: a practical and powerful approach to multiple testing. J R Stat Soc Series B Stat Methodol. 1995;57(1):289-300.
20. Cohen J. A coefficient of agreement for nominal scales. Educ Psychol Meas. 1960;20(1):37-46.
21. Landis JR, Koch GG. The measurement of observer agreement for categorical data. Biometrics. 1977;33(1):159-74.
