# Supplementary material for: Depictions of Depression in Generative AI Video Models: Mixed Methods Study of OpenAI’s Sora 2
Source: J Med Internet Res. 2026 Sep 14;28:e95682. doi: 10.2196/95682 (PMC13574309; doi:10.2196/95682)
Supplement: Supplementary file 5 [file jmir-v28-e95682-s005.docx]

**Appendix E: Inter-Rater Reliability Results**

Two coders independently coded all 100 videos. The primary coder's data (MF) was used for all analyses; the second coder's independent coding (ZZ) was used to calculate reliability.

**Narrative Arc**

The three-way classification (recovery, deterioration, no shift) showed poor agreement on deterioration, so we collapsed to a binary classification of recovery vs. no recovery (no shift + deterioration), which achieved substantial agreement (κ = 0.71, 86% overall agreement). When both coders identified narrative shifts, temporal agreement was strong (mean difference = 0.31 seconds, median = 0.00 seconds, n = 35 pairs).

**Environments**

Overall presence/absence agreement was excellent (κ = 0.91) with strong temporal agreement (mean IoU = 0.84). Category-specific kappas ranged from substantial to perfect:

| **Category** | **κ** | **Agreement** |
| --- | --- | --- |
| Bathroom | 1.00 | 100% |
| Kitchen/Dining | 1.00 | 100% |
| Elevated Outdoor | 0.97 | 99% |
| Urban Outdoor | 0.97 | 99% |
| Underwater | 0.94 | 99% |
| Transit | 0.91 | 98% |
| Natural Outdoor | 0.89 | 97% |
| Bedroom | 0.82 | 96% |
| Other Interior | 0.78 | 94% |
| Abstract | 0.66 | 98% |
| At Window | 0.53 | 90% |

**Objects**

Overall presence/absence kappa was moderate (κ = 0.61) with strong temporal agreement when objects were mutually identified (mean IoU = 0.82). Agreement varied substantially by object type.

*High reliability objects* (κ > 0.70): mug/cup (κ = 0.93), pen (κ = 0.90), mirror (κ = 0.90), bench (κ = 0.86), birds (κ = 0.81), lamp (κ = 0.81), cityscape/buildings (κ = 0.80), cloud (κ = 0.77), plant/flowers (κ = 0.71).

*Lower reliability objects*: bed (κ = 0.44), window (κ = 0.47), sweatshirt/hoodie (κ = 0.29), rain (κ = 0.23). Examination of disagreements revealed an asymmetric pattern: the primary coder noted these ubiquitous items more exhaustively, while the secondary coder focused on more distinctive elements. This asymmetric pattern suggests the lower agreement values partly reflect a threshold difference in tagging exhaustiveness, though the resulting kappa values should be interpreted with this limitation in mind.

**Figure Demographics**

| **Variable** | **κ** | **Agreement** |
| --- | --- | --- |
| Gender | 1.00 | 100% |
| Age | 0.38 | 89% |
| Race/Ethnicity | 0.53 | 68% |
| Apparent SES | 0.20 | 56% |

Gender achieved perfect agreement. Age showed high raw agreement (89%) but low kappa (κ = 0.38) due to restricted range (88% of figures coded as 20-30). Race/ethnicity showed moderate agreement (κ = 0.53), likely due to numerous bi-racial and ethnically ambiguous AI-generated figures; this dimension was excluded from analysis. Apparent SES showed poor agreement (κ = 0.20) and was excluded from further analysis.

**Figure States**

| **Variable** | **κ** | **IoU** |
| --- | --- | --- |
| Posture | 0.89 | 0.86 |
| Alone in Frame | 0.94 | 0.98 |
| Facial Expression | 0.49 | 0.63 |

Posture and alone-in-frame status achieved strong reliability. Facial expression showed poor agreement (κ = 0.49) due to ambiguous AI-generated expressions and was excluded from analysis.

**Gaze Direction**

Coders frequently noted directional gaze in free-text annotations. When both coders documented gaze direction, agreement on dominant direction was high (96%, n = 84). The primary coder noted downward gaze more frequently (93 videos) than the secondary coder (79 videos), reflecting threshold differences in annotation.

**Dimensions Excluded from Analysis**

Based on reliability results, the following dimensions were excluded from substantive analysis: race/ethnicity (κ = 0.53), apparent SES (κ = 0.20), and facial expression (κ = 0.49).
